# When Estrogen Signaling Refuses to Die: Receptor Rewiring, Compartmentalization, and Endocrine Plasticity in Gynecological Cancers

**DOI:** 10.3390/ijms27041924

**Published:** 2026-02-17

**Authors:** Jimena P. Cabilla, María Teresa L. Pino

**Affiliations:** Centro de Altos Estudios en Ciencias Humanas y de la Salud, Consejo Nacional de Investigaciones Científicas y Técnicas (CONICET), Universidad Abierta Interamericana, Buenos Aires C1270AAH, Argentina; jimena.cabilla@uai.edu.ar

**Keywords:** cervical cancer, ovarian cancer, endometrial cancer, estrogen receptor alpha, estrogen receptor beta, GPER1, cell signaling

## Abstract

Although estrogen signaling plays an important role in gynecological cancers, its function is highly context-dependent and often contradictory. Estrogen receptors have been associated with both tumor-promoting and tumor-suppressive effects depending on the tumor type, disease stage, and cellular environment. This review summarizes the current evidence on estrogen receptor signaling in cervical, ovarian, and endometrial cancers, focusing on receptor subtype balance, isoform diversity, cellular and subcellular localization, and epigenetic regulation. Rather than a static marker, estrogen receptor expression is revealed as a dynamic and plastic signaling network. In cervical cancer, estrogen signaling persists despite the loss of epithelial estrogen receptor α (ERα) through stromal signaling, alternative ERα isoforms, ERβ, and non-classical receptors such as G protein-coupled estrogen receptor 1 (GPER1). In ovarian cancer, epigenetic silencing of ERβ and ERα predominance drives oncogenic signaling while also creating specific biological vulnerabilities. In endometrial cancer, estrogen signaling shifts from hormone-dependent initiation to progressive oncogenic autonomy through receptor rewiring and non-genomic pathways. By integrating these mechanisms, this review highlights estrogen receptor plasticity as a unifying concept across gynecological malignancies and outlines key knowledge gaps that are relevant for future endocrine strategies.

## 1. Introduction

Estrogen signaling plays a central role in the physiology of the female reproductive system and in the development and progression of gynecological malignancies [1,2]. Although estrogen receptors have been studied extensively for decades, their functions in cancer remain paradoxical and highly context-dependent [3,4]. Depending on receptor subtype, isoform expression, subcellular localization, cellular composition, and microenvironmental cues, estrogen signaling may drive proliferation, differentiation, invasion, or therapeutic resistance [5,6,7,8].

These divergent outcomes cannot be explained by receptor expression alone. Accumulating evidence indicates that estrogen responses arise from a dynamic and adaptable signaling network involving receptor crosstalk, non-genomic pathways, epigenetic mechanisms, and non-coding RNA regulation [2,9,10]. This complexity has generated apparently conflicting observations across tumor types, disease stages, and experimental models, underscoring the need for an integrative conceptual framework [8,11,12].

In this context, the present review synthesizes the current knowledge on estrogen receptor biology and signaling in gynecological malignancies. The structural and functional features of estrogen receptors and their major signaling modalities are first addressed, followed by an integrated overview of estrogen receptor expression and dynamics in normal gynecological tissues across the menstrual cycle and menopause. Building on this physiological framework, tumor-specific patterns of estrogen receptor plasticity in cervical, ovarian, and endometrial cancers are examined, with an emphasis on context-dependent signaling and microenvironmental interactions. Finally, the contribution of non-coding RNAs to estrogen receptor regulation is discussed as a key layer shaping estrogen signaling in gynecological cancer.

## 2. Estrogen

Estrogens are a group of steroid hormones that, together with progesterone, constitute the main female sex hormones. There are both endogenous and exogenous estrogens. Endogenous estrogens are secreted by glands or cells in living organisms and include phytoestrogens found in plants (such as isoflavones, coumestans, and lignans) [13]. In women, estrogens are responsible for the development of primary and secondary sexual characteristics. Their main function is to stimulate cellular proliferation and growth in reproductive tissues. They promote the development of mammary tissue from puberty and play an essential role in regulating the menstrual cycle. However, in both women and men, estrogen has a broad range of physiological functions, such as regulation of bone mineralization, cholesterol mobilization, brain and metabolic functions, inflammation control, and roles in the cardiovascular and immune systems [14]. The primary estrogen-producing organ is the ovary, specifically granulosa cells, although estrogens can also be produced in the adrenal gland, testes, placenta, adipose tissue, liver, pancreas, and others.

Humans synthesize four different types of estrogens, which bind both nuclear and membrane receptors with varying affinity and trigger different cellular responses. These are: estrone (E1), present in high levels during menopause; estradiol (E2 or 17β-estradiol), predominantly synthesized by the ovaries in premenopausal women; estriol (E3), secreted by the placenta during pregnancy; and estetrol (E4), also synthesized during pregnancy in the fetal liver and transferred to the mother via the placenta [15]. However, the term “estrogen” generally refers to E2 due to its physiological importance and wide distribution in the human body, and it will therefore be used as such throughout this work.

E2 exerts its functions through interaction with estrogen receptors (ERs). There are three types of ERs: the nuclear receptors ERα and ERβ and a G protein-coupled membrane receptor called GPER1 (also known as GPER or GPR30).

## 3. Estrogen Receptors

### 3.1. Estrogen Receptor Alpha (ERα)

Among ERs, the most studied is ERα, a member of the nuclear receptor superfamily, thus primarily acting as a ligand-inducible transcription factor. Of all the estrogen receptors, ERα has been the most studied and largely associated with E2 tumor-promoting actions [16,17,18].

**Figure 1:**
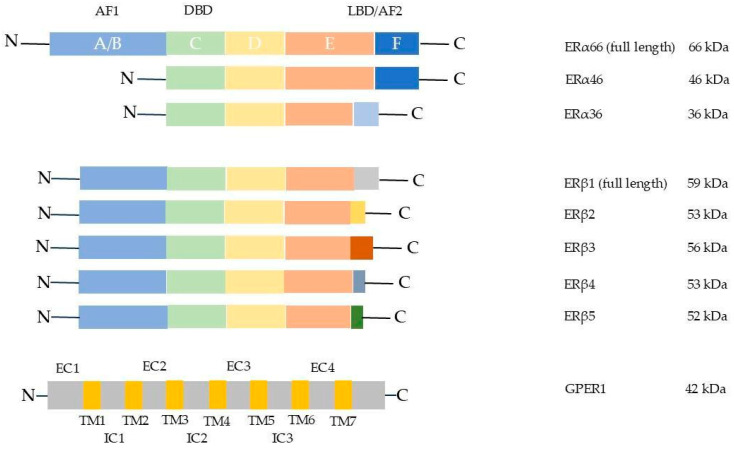
The main estrogen receptors and their isoforms. Cytoplasmic/nuclear receptors ERα and ERβ are mostly organized into 5 domains: activation-function 1 (AF-1), DNA-binding domain (DBD), a hinge region, ligand-binding domain (LBD), and AF-2. The most conserved regions among all receptors are C, D, and E. Membrane-bound GPER1 receptor encompasses 4 extracellular domains (EC1-4), 3 intracellular domains (IC1-3), and 7 transmembrane domains (TM1-7).

Upon estrogen binding, ERα plays a key role in regulating gene transcription, not only of genes yielding estrogen response elements (EREs) but also of other genes lacking EREs through the recruitment of co-adapters. The *ESR1* gene, located on chromosome 6 (6q25.1), encodes estrogen receptor alpha (ERα). Structurally, several functionally related domains have been identified. Region C is a zinc finger-containing domain involved in DNA binding. Regions A/B located at the N-terminal portion of ERα include the activation function 1 (AF-1) domain, which mediates ligand-dependent as well as ligand-independent activation. Region D, identified as the hinge region, lends flexibility to the protein structure, whereas region E, the ligand-binding domain (LBD), not only activates AF-2 in a ligand-dependent manner but is also involved in receptor dimerization and interaction with both coactivators and corepressors. In contrast, the function of Region F is not yet well described [16,19]. Different isoforms of ERα are products of gene splicing (Figure 1). In addition to the ERα full-length receptor (wild type), two ERα isoforms, ERα-46 and ERα-36, have been identified. Both receptors lack the AF-1 domain, whereas ERα-36 also lacks the AF-2 domain [20].

### 3.2. Estrogen Receptor β (ERβ)

Estrogen receptor beta (ERβ) is encoded by the *ESR2* gene located on chromosome 14q23.2. Structurally, ERβ shares with ERα the characteristic modular organization of nuclear receptors, consisting of six domains (A–F) [21]. In comparison to ERα, ERβ exhibits notable sequence divergence within the N-terminal AF-1 and C-terminal F domains, which accounts for their distinct transcriptional profiles and biological effects [22]. Five splicing isoforms have been described so far, all of them shielding shorter LBD and no AF-2 domain function, resulting in no estrogen-binding ability (Figure 1). Only the full-length isoform ERβ1 is able to bind estrogen ligands [5]. The main differences among ERβ isoforms are summarized in Table 1.

ERβ displays a broad and heterogeneous tissue distribution distinct from ERα. While ERβ is not predominantly expressed in any tissue, it is abundantly expressed in the ovary, particularly in granulosa cells, playing an essential role in follicular development. High levels are also found in the prostate, cardiovascular and central nervous systems, among others [27]. Its wide tissue distribution indicates that ERβ mediates both reproductive and non-reproductive functions of estrogens, often in contexts where ERα is absent or expressed at lower levels.

Functionally, ERβ is a versatile regulator of gene expression and cellular signaling. Upon ligand binding, ERβ can homodimerize or heterodimerize with ERα, and the composition of these dimers determines the transcriptional outcome. In a very general overview, which will be discussed throughout this review, ERβ is considered to exert antiproliferative, pro-differentiation, and anti-inflammatory effects, which contrast with the more proliferative actions associated with ERα. In reproductive tissues, ERβ regulates folliculogenesis, ovulation, and ovarian hormone production. Collectively, these diverse functions underscore ERβ’s role as a key mediator of E2 protective and regulatory actions across multiple organ systems.

### 3.3. G Protein-Coupled Estrogen Receptor (GPER1)

G protein-coupled estrogen receptor 1 (GPER1, formerly known as GPR30) is encoded by the *GPER1* gene located on chromosome 7p22.3. It is a seven-transmembrane-domain receptor belonging to the G protein-coupled receptor (GPCR) superfamily and is structurally unrelated to the nuclear estrogen receptors ERα and ERβ. It was first cloned as an orphan receptor until it was later described as an E2-binding receptor. Unlike these nuclear receptors, GPER1 is primarily localized at the plasma membrane and intracellular compartments such as the endoplasmic reticulum, where it mediates rapid non-genomic signaling in response to E2 [28]. GPER1 is widely expressed across diverse tissues, including the ovary, testis, endometrium, and placenta, among others [29]. Functionally, activation of GPER1 by E2 or selective agonists initiates multiple intracellular signaling cascades, including adenylyl cyclase stimulation, intracellular calcium mobilization, and activation of the MAPK and PI3K/AKT pathways [29,30,31]. Through these mechanisms, GPER1 regulates a wide array of physiological processes, such as reproductive function, cardiovascular and bone homeostasis, metabolic regulation, immune modulation, and neuroprotection, among others [32]. Furthermore, aberrant GPER1 signaling has been increasingly associated with pathological conditions, including cancer progression, metabolic and reproductive dysfunctions, immune-related disorders, and cardiovascular disease, underscoring its importance as a pivotal mediator of the rapid non-genomic actions of E2 [7,33]. In contrast to ERα and ERβ, functional protein isoforms of GPER1 have not yet been formally described.

The structure and diversity of ERα, ERβ, and GPER1 are depicted in Figure 1.

## 4. Expression and Dynamics of Estrogen Receptors in Normal Gynecological Tissues

The homeostasis of female reproductive tissues is regulated by a plastic estrogen signaling network that adapts to hormonal fluctuations during the menstrual cycle and the transition to menopause. This network is mediated by the nuclear receptors ERα and ERβ and the membrane-associated receptor GPER1 [34,35].

### 4.1. Cervix and Uterus

In the cervix and uterus, ERα expression fluctuates across the menstrual cycle and decreases markedly following the cessation of ovarian function [36]. In contrast, ERβ is constitutively expressed in the nucleus and cytoplasm of histologically normal cervical and uterine epithelium, predominantly in the basal and parabasal layers [36]. Importantly, ERβ expression in parabasal cells was regulated by the menstrual cycle: positive in the follicular phase (proliferative phase) and negative in the luteal phase (when progesterone receptor is positive) [37]. A colocalization of ERβ with leukocytic markers has also been reported in the human cervix [36].

Also, ERβ mRNA levels are significantly higher in the myometrium of postmenopausal women compared with premenopausal women [38], suggesting that residual estrogen signaling during menopause is preferentially mediated through ERβ to support epithelial homeostasis rather than cyclic proliferation [36].

### 4.2. Ovary

In the normal human ovary, estrogen receptor expression is functionally segregated. ERα is detected in the thecal cells and the germinal epithelium, compartments that are essential for steroidogenesis and ovarian surface integrity, respectively [34]. Conversely, ERβ shows complementary localization and is predominantly expressed in granulosa cells and ovarian follicles [9,34], supporting compartmentalized regulation of follicular physiology [9]. ERβ, including its splice variants, is the dominant estrogen receptor in normal ovarian tissue and is essential for ovarian function and female fertility [38,39,40,41,42,43,44,45], with an ERα:ERβ ratio of approximately 1:9 in humans [45]. ERβ is mainly localized in granulosa cell nuclei, with additional expression in perithecal and stromal cells [9,41,46]. High ERβ expression in granulosa cells is critical for cell differentiation and the ovulatory response to gonadotropins, processes that are indispensable for fertility [46,47,48].

### 4.3. Endometrium

The endometrium is a hormone-responsive tissue whose physiological maintenance—characterized by cyclic proliferation, secretion, and desquamation—depends on the temporospatial regulation of ERα, ERβ, and GPER1 [49,50]. ERα is the dominant receptor during the proliferative phase, showing stronger immunoreactivity than ERβ in epithelial, stromal, and myometrial nuclei, with the mRNA and protein levels peaking at the end of the proliferative phase and early secretory phase before declining thereafter [50,51,52]. ERα is expressed in epithelial and stromal compartments and is also detected in uterine arterial smooth muscle cells [50,53]. In contrast, ERβ mRNA levels remain low during proliferation but peak at the end of the secretory phase, becoming the predominant subtype in the endometrial stroma [50,52]. Although ERβ is expressed in glandular and stromal nuclei, endometrial endothelial cells exclusively express ERβ, indicating a specialized role in mediating selective E2 effects within the uterine vasculature [50,52]. ERβ mRNA levels are also significantly higher in the myometrium of postmenopausal women [36]. GPER1 is expressed in epithelial, stromal, myometrial, and decidual cells, with maximal expression in glandular epithelial cells during the mid-to-late proliferative phase and rapid downregulation at the onset of the secretory phase [50]. GPER1 is predominantly localized to the plasma membrane and cytoplasm of epithelial cells, consistent with its role in mediating non-genomic E2 signaling [50,54,55]. The expression patterns and functional dynamics of estrogen receptors in normal gynecological tissues during the menstrual cycle and after menopause are summarized in Table 2.

## 5. Role of Estrogen Receptors in Gynecological Cancers

Estrogen receptors (ERα, ERβ, and GPER1) are key mediators in gynecological carcinogenesis that, depending on the tissue or the tumor microenvironment, can have a protumoral effect or, in certain contexts, a tumor-suppressive role [9,56,57]. Here, we discuss the reported evidence regarding the role of ERs in cervical, endometrial, and ovarian cancer. In this review, we use the term “estrogen receptor plasticity” to describe the dynamic integration of receptor expression patterns, subcellular localization, signaling pathway selection, and microenvironmental cues that collectively shape estrogen responses in gynecological cancers.

### 5.1. Cervical Carcinoma

#### 5.1.1. Epidemiology and Hormonal Contributions in Cervical Carcinoma: When HPV Meets Estrogen

Approximately 660,000 new cases and 350,000 deaths worldwide in 2022 position cervical carcinoma (CC) as a leading cause of cancer-related death among women in 36 countries [58,59]. CC comprises HPV-dependent and HPV-independent entities with distinct pathogenic and biological characteristics [60]. While most CCs are HPV-driven, a subset of cervical adenocarcinomas (CACs) arises independently of HPV and displays different genetic profiles, including KRAS mutations [8,61,62]. This review is mainly focused on HPV-driven CC.

Although persistent infection with high-risk HPV represents the major cause of CC, epidemiological evidence points to exposure to E2 as a necessary partner in its development [61,62] since exogenous hormonal factors, such as long-term use of oral contraceptives [63] and number of full-term pregnancies, have been consistently associated with an increased risk of CC in women infected with HPV [64,65].

Building on these epidemiological observations, investigations conducted with transgenic mice expressing HPV-16 oncogenes have been crucial in establishing the relationship between cervical cancer, E2, and its receptor ERα [66,67,68]. Using this model, Arbeit et al. and Elson et al. initially demonstrated that exogenous exposure to E2 was necessary to efficiently induce the cervical squamous epithelium to neoplastic progression, indicating that E2 acts as a tumor promoter in collaboration with viral oncogenes [67,68].

Expanding on these findings, Chung et al. studied the involvement of the E2 pathway and its main receptor, ERα, in the development of CC. The investigators generated HPV-transgenic mice that were also ERα-deficient (ERα knockout, -/-) and found that, while ERα-expressing mice developed CC or precursor lesions (cervical intraepithelial neoplasia, CIN), ERα-deficient ones failed to develop any stage of this neoplastic progressive disease after exposure to E2 [62]. This finding established that signaling through ERα is a fundamental requirement mediating the carcinogenic effects of E2 in HPV-associated cervical carcinogenesis [62,69].

#### 5.1.2. ERα in Cervical Carcinogenesis: Essential, Paradoxically Downregulated

Despite this clear ERα dependence, histopathological studies of human cervical lesions—from CIN to invasive carcinoma—have shown a downregulation or even loss of ERα in malignant epithelial cells and maintenance of ERβ expression [34,35,69,70,71,72]. Nikolaou et al. quantified this reduction after analyzing ERα positivity in normal squamous CC (CIN) and invasive squamous CC (SCC) samples and found that ERα positivity significantly decreased from 31.15% in CIN to 11.15% in SCC [35]. These results tally with those of Singh et al., who found ERα positivity in only 6.67% of analyzed CC cases in contrast to 90% found in controls [72].

Interestingly, López-Romero et al. detected ERα mRNA transcripts in 90% of invasive carcinomas but not the protein, suggesting that ERα loss could occur through post-transcriptional and/or post-translational mechanisms during invasive carcinoma progression [34]. Furthermore, ERα loss was associated with greater aggressiveness: Zhai et al. demonstrated that ERα knockdown increased CC cell invasion capability, suggesting an invasion-suppressive role for ERα [34,73].

Together, these findings create a paradox: if ERα-mediated signaling is necessary for tumor progression, how can a tumor progress when ERα is being downregulated in CC cells? To address this paradox and fully understand the underlying mechanism, Chung et al. hypothesized that essential ERα was not located in the epithelium where the malignant lesion resides but in the surrounding microenvironment—the stroma [74,75]. In their study, HPV-transgenic mice were exposed to E2 for 6 months until they developed cervical dysplasia or invasive carcinoma. Once established, stromal ERα was temporally and selectively deleted through a tamoxifen-inducible Cre recombinase system, causing complete regression of cervical dysplasia.

Moreover, many other reports have validated this ER dependence by using selective estrogen receptor modulators (SERMs). In this sense, raloxifene and fulvestrant were shown to prevent and even treat already established cervical lesions in HPV-associated murine models [1,62,68,70,76,77]. However, Spurgeon et al. showed that treatment with raloxifene, an ERα antagonist, was an effective therapy that initially induced disease regression. Nevertheless, once raloxifene treatment was discontinued, dormant cancer cells resumed their growth program, leading to tumor reactivation [77,78].

Strikingly, Nair and Luthra found that the aromatase enzyme was expressed in situ in 35% of analyzed CCs, indicating an intrinsic capacity for E2 production within the tumor microenvironment [79]. In addition, Tomaszewska et al. showed that circulating estrone (E1) was converted to E2 by 17β-hydroxysteroid dehydrogenase 1 (17β-HSD1), an enzyme that was found to be overexpressed not only in CC cell lines, such as HeLa, SiHa, CaSki, and C-33 A, but also in human primary CC. These overexpressed protein levels were found even when the mRNA levels were similar between normal and cancerous tissues, suggesting post-transcriptional regulation of this conversion [80].

These findings might explain why SERMs may induce remission but not prevent relapse since stromal ERα activity and locally synthesized E2 may cooperate to sustain tumor growth and progression, even in the absence of epithelial ERα expression. Collectively, these results highlight the fundamental role of ERα and its tight paracrine relationship with tumor growth [74,75].

In contrast to HPV-associated squamous lesions, HPV-independent cervical carcinomas, particularly CACs, display distinct patterns of ER expression. In HPV-independent models such as C-33 A, functional expression of ERα, ERβ, and GPER1, together with local E2-metabolizing capacity, have been reported, suggesting estrogen responsiveness in the absence of viral oncogenic drivers [4,80]. Clinically, HPV-independent CAC is often diagnosed at more advanced stages [60] and commonly retains ERα and progesterone receptor expression, a feature that has been associated with improved survival in some studies, whereas high GPER1 expression has been linked to increased invasiveness and poorer prognosis [8,81,82].

#### 5.1.3. ERα-36: The Alternative Driver in Cervical Tumor Progression

Whereas ERα is downregulated in HPV-driven CC, ERα-36 has been shown to be upregulated. This splicing variant of the *ESR1* gene was overexpressed mainly in plasma membrane and cytoplasm, not only in CC cell lines (SiHa, HeLa, CaSki, and C-33 A) but also in tissues from CC patients. This subcellular distribution contrasts with the classical nuclear localization of ERα [6,10,83]. In tumor tissues, Wang et al. (2021) showed that ERα-36 levels were significantly higher—48.5% in squamous cell carcinoma (SCC) and 55.6% in CAC—than in CIN (30%) and normal tissues (13.3%) [83].

Sun et al. demonstrated that ERα-36 subcellular localization was directly associated with the activation of the MAPK/ERK cascade in CC cell lines (CaSki and HeLa) after E2 stimulation, relating this cell compartmentalization to cell proliferation and metastasis [6,10,83]. This mechanism has also been reported in EC, where ERα-36 activates PKCδ/ERK, confirming the functional conservation of this pathway [84,85].

Several functional assays have demonstrated that ERα-36 acts as an oncoprotein in CC. Lentiviral-mediated ERα-36 overexpression promoted CaSki and HeLa cell proliferation after 1 nM E2 stimulation. Conversely, ERα-36 silencing suppressed E2-driven proliferation [6,83]. Moreover, ERα-36 silencing induced cell cycle arrest in the G0/G1 phases [6]. Furthermore, ERα-36 was shown to promote migration and invasion as its silencing abrogated these effects [6,83]. Likewise, in vivo xenogeneic studies confirmed that ERα-36 overexpression was associated with increases in tumor volume and Ki67 proliferation index, thus validating ERα-36’s oncogenic role under physiological conditions [6,83].

Based on next-generation sequencing (NGS) analysis, the high-mobility-group A2 protein (HMGA2) was identified as a downstream target of ERα-36 [83]. Moreover, HMGA2 silencing attenuated the oncogenic effects of ERα-36, showing that this ERα-36-upregulated protein is a crucial effector of the E2-mediated proliferation and metastasis cascade [83].

In the CC of HPV etiology, a remarkable mechanism involves the interaction of ERα-36 with viral oncogenes. Zhang et al. found that ERα-36 upregulates E6 and E7 at the mRNA and protein levels, whereas ERα acts as a negative regulator [86]. ERα-36-driven stimulation was associated with p53, p21, and cyclin D1 deregulation, thereby affecting cell cycle control. It was also found that ERα-36-mediated cell migration and invasion were linked to activation of the Wnt/β-catenin/MRTF-A pathway, which is dependent on HPV E7 oncoprotein [86].

ERα-36 expression has also been linked to tumor aggressiveness and adverse prognosis in CC beyond its molecular effects [83]. It was correlated with reduced overall survival in SCC and CAC and identified as an independent predictor of poor survival in SCC by multivariate Cox regression analysis [83]. Moreover, increased levels of ERα-36 were significantly associated with advanced FIGO stage, deep stromal invasion (DSI), lymph node metastasis (LNM), and high Ki67 expression [83]. Co-expression of ERα-36 and HMGA2 was linked to an even worse prognosis, suggesting that the combined evaluation of both markers may serve as a more sensitive prognostic tool [83].

In summary, identifying the opposite roles of ERα isoforms is essential to develop directed therapeutic strategies and broaden the possibility of designing specific therapies to restore the balance between ERα isoforms, thereby limiting tumor progression and promoting a better prognosis.

#### 5.1.4. ERβ in Cervical Cancer: From Persistence to Metabolic Adaptation

As discussed above, ERα loss in malignant epithelium is an early and consistent event, whereas ERβ continues to be expressed [34,72,87].

Accumulated evidence demonstrates that ERβ expression is conserved and stable throughout cervical carcinogenesis. In invasive squamous carcinomas, ERβ was detected in 70–80% of the samples, and the ERβ mRNA levels showed comparable positivity in these tumors [34,70,72]. Likewise, there were no significant differences in ERβ immunoreactive positivity between non-neoplastic cervical tissues and tumors [72]. In this line, López-Romero et al. found that ERβ expression remained conserved from normal cervical epithelium to invasive carcinoma, including low-grade squamous intraepithelial lesions (LSILs) and high-grade squamous intraepithelial lesions (HSILs) [87].

Intriguingly, ERβ cell localization changes as malignancy progresses since ERβ is predominantly located in the nuclei of normal cervical epithelial cells, but, in invasive CC, immunoreactivity was nuclear and cytoplasmic in the invasion areas [34]. The authors hypothesized that this change in cell localization could be related to the expression of specific ERβ isoforms (ERβ2 or ERβ5), which in other contexts is related to metastases and worse prognoses. However, this discrimination between ERβ isoforms was not tested in this study [34].

Aside from ERβ changes in expression and localization, a mechanism explaining its persistence in neoplastic epithelium also involves the participation of the epigenetic factor BORIS (brother of regulator of imprinted sites, also known as CTCFL). BORIS was reported to be absent in normal cervical epithelium but to increase as the lesion progresses toward invasive squamous carcinoma, showing a positive association with malignant progression [87]. Additionally, López-Romero et al. demonstrated a strong correlation between BORIS and ERβ at both the mRNA and protein levels in clinical samples and cell lines regardless of HPV genotype. This co-expression suggests that BORIS functions as a transcriptional activator that maintains ERβ expression during cervical carcinogenesis, thereby contributing to the persistence of estrogenic signaling in tumor cells [87].

An additional mechanism linking estrogenic signaling with invasiveness in CC involves Ezrin, a key protein in the ERM (Ezrin–Radixin–Moesin) family that is highly relevant for cytoskeleton reorganization and cell motility [70]. Ezrin was shown to be induced by E2 in CC cell lines, causing its translocation to cell peripheries and promoting pseudopodal formation and membrane curling, thereby contributing to an invasive phenotype. Treatment with tamoxifen blocked Ezrin induction and E2-mediated morphological changes, demonstrating that the ERs were able to induce Ezrin-mediated invasiveness in CC cell lines. IHC analysis of biopsies from patients classified from normal tissues to invasive SCC showed that Ezrin expression increased from intraepithelial lesions to invasive carcinoma, whereas ERα and ERβ gradually decreased during malignant progression [70].

The paradox here is: if Ezrin upregulation depends on ER signaling in CC cell lines, how can Ezrin levels increase while its upstream mediators decrease in patient tissues? The authors see this apparent contradiction as being caused by an oncogenic bypass triggered by HPV. Viral oncoproteins E6/E7 induce the transcription factor SIX1 (Sine oculis-related homeobox 1 homologue), which upregulates Ezrin independently of ER, promoting invasiveness even when ER levels are reduced. However, functional studies show that residual ER can still promote changes in the cytoskeleton and Ezrin-dependent movement, implying that estrogen signaling might still support invasion as the HPV–SIX1 viral pathway becomes more dominant in advanced stages of the disease [70].

Another discrepancy regarding ERβ tissue expression in CC represents an important point of interest in the literature. While some authors report a progressive decrease in ERβ as the disease advances, others indicate that this subtype remains expressed and functional in a substantial proportion of invasive CC cases [34,70,72,87]. This divergence has been attributed mainly to methodological factors, especially the heterogeneity of the antibodies used for IHC detection, which differed in the epitopes they recognize and therefore in their ability to differentiate between the multiple ERβ isoforms (ERβ1–5) and their subcellular localization patterns [34,70]. Therefore, reports describing a “loss” of ERβ may probably indicate the absence of specific isoforms rather than a true reduction in its expression [34,70]. Despite these technical limitations, the conclusion remains consistent: ERβ acts as an important mediator of estrogenic signaling in neoplastic epithelium, although, in advanced stages of the disease, its influence becomes less significant as the HPV-driven oncogenic mechanisms assume a predominant role, particularly the activation of SIX1 and the subsequent induction of Ezrin through ER-independent pathways [70].

As ERβ is expressed in neoplastic cells [34,72], many hypotheses have been proposed for its role in CC. One of these hypotheses attributes an important role to ERβ in invasion and metastasis. Metalloproteases (MMPs) such as MMP9, MMP10, MMP11, and MMP12 have been well documented in CC [34,88]. Additionally, MMP2 and MMP9 have been shown to be activated by E2 in other pathological contexts [89]. These observations raise the possibility that ERβ might be involved in the regulation of these proteases in CC epithelia. In this sense, evidence points to ERβ-mediated non-genomic pathways based on the fact that ERβ is located mainly in cell cytoplasm in the tumor invasive zones [34,35,90]. Also, this cytoplasmic pattern has already been associated with a worse prognosis in other tumors, such as breast and vulvar tumors [90,91,92]. The presence of ERβ in the cytoplasm opens the possibility that it modulates rapid signaling factors. One of these is AP-1, whose recruitment can promote MMP9 transcription and consequently favor the invasive capacity of the tumor to infiltrate surrounding tissues [34,93]. Altogether, ERβ could be related to invasion in CC. However, some reports have also related ERβ to metabolic adjustments, more specifically to the Warburg effect.

Although ERα and ERβ are commonly found together in the mitochondria of CC cells, Liao et al. assign ERβ a leading role in malignant metabolic adaptation as they found that ERβ directly interacts with mitochondria, regulating cellular bioenergetics at the mitochondrial DNA (mtDNA) level [94]. It was demonstrated that mtDNA contains ERE-like sequences that can bind recombinant ERα and ERβ, and that this binding is intensified in the presence of E2 [69,95]. Through this mechanism, ERβ contributes to the regulation of the respiratory chain, and to new mitochondrial biogenesis [96,97]. Hence, retaining functional ERβ in CC allows it to respond to estrogenic signals and to control internal cellular energetic machinery. E2-mediated ERβ activation promotes the Warburg effect, a metabolic program that helps tumor cells to satisfy high energetic and biosynthetic demands [69]. Studies performed in CC cell lines demonstrated that E2-exposed SiHa and HeLa cells increase glucose consumption and lactic acid release into culture media, both of which are clear metabolic signatures of the Warburg effect. The Warburg effect is widely accepted to be a consequence of dysfunctional oxidative metabolism, and E2 not only induces mitochondrial dysfunction in CC cells but also upregulates key glycolysis and gluconeogenesis genes such as LDHA, LDHB, PGK1, and TPI1 and downregulates PFKP. All these upregulated genes are necessary for maintaining glycolytic fluxes, but it was also shown that E2 differently regulates 45 genes involved in oxidative phosphorylation, thereby affecting mitochondrial respiratory complex activities. In summary, E2 and ERβ–mitochondria direct interaction gives invasive CC the ability to survive under the inherent metabolic stress of tumorigenesis [69].

In summary, the overall evidence shows that ERβ not only persists but also remains an important factor in the biology of CC, promoting invasion and metabolic adaptation even when HPV oncogenic pathways begin to dominate. All these findings suggest that the estrogenic response in tumors is more complex than just ERα loss or ERβ persistence, prompting consideration of other mediators, such as GPER1.

#### 5.1.5. GPER1: A Double Agent in Cervical Carcinoma

GPER1, a receptor frequently found in CC, mediates E2 rapid action [3,4,8]. In CC, GPER1 function is notably contrasting, showing both tumor-promoting and tumor-suppressive roles [3,4,8,98].

The functional evidence suggesting a tumor-suppressive role of GPER1 is mainly in vitro, showing that G-1-induced GPER1 activation inhibited the proliferation of CC cell lines [3,99]. This antiproliferative effect was achieved by the sustained activation of ERK1/2, leading to a dysregulation of cyclin B and thus inducing cell cycle arrest in G2/M phases [3,4,56,99]. Also, GPER1 stimulation induced diverse cell death programs, including apoptosis and senescence in HeLa, SiHa and C-33 A cell lines [3,56]. Moreover, GPER1 knockdown increased the ability of HeLa and SiHa cells to form colonies (in size and/or number) and enhanced invasiveness, an indicator of stemness properties. Immunofluorescence staining showed strong GPER1 expression at the peripheries and sprouts of cell tumorspheres (invasive front), in concordance with an active role in modulating tumor behavior. Furthermore, GPER1 knockdown increased HeLa EMT by augmenting the cellular long-to-wide ratio and the formation of longer filopodia, both of which are characteristics of motility and metastasis. Additionally, in HeLa and SiHa cell lines, GPER1 knockdown led to a significant increase in the oncogene SERPINE1/PAI − 1, a factor associated with a worse prognosis in CC [4].

**Figure 2:**
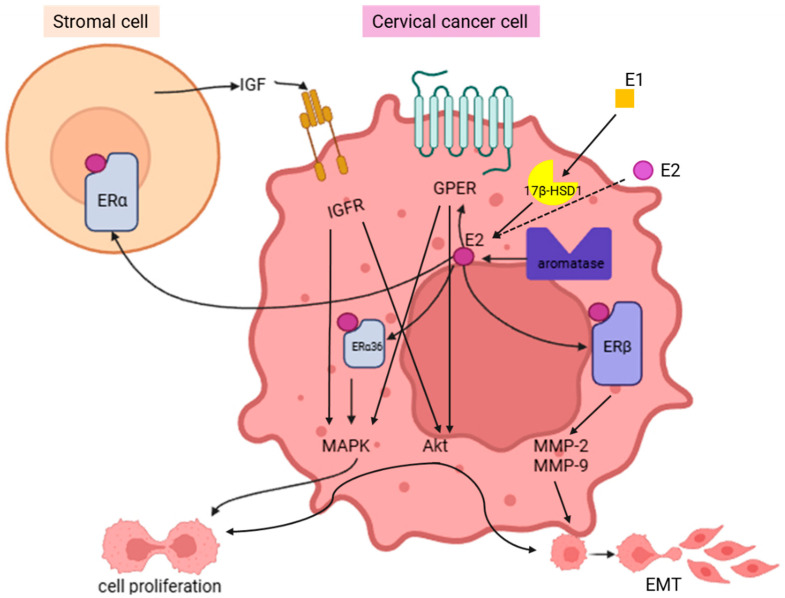
Role of estrogens and ERs in CC. Most CC cells lose expression of ERα but maintain high levels of ERα-36, G protein-coupled estrogen receptor 1 (GPER), and ERβ. In contrast, ERα is found in tumor-associated stromal cells. CC cells produce aromatase and 17β-hydroxysteroid dehydrogenase type 1 (17β-HSD1), allowing for local synthesis of 17β-estradiol (E2). E2 can activate all ERs through autocrine and paracrine signaling. In stromal cells, ERα mediates estrogen responses and promotes the secretion of insulin-like growth factor (IGF), which then activates IGF receptor (IGFR) signaling in nearby CC cells. In CC cells, E2 activates multiple receptors, including GPER, ERα-36, and ERβ. These pathways converge on downstream cascades such as MAPK and Akt, promoting cell proliferation. Additionally, ERβ signaling induces the expression of matrix metalloproteinases (MMP-2 and MMP-9), facilitating extracellular matrix remodeling, epithelial-to-mesenchymal transition (EMT), and tumor cell invasion. Figure created with BioRender.

The role of estrogen and ERs in CC is summarized in Figure 2.

Despite the evidence supporting the tumor-suppressive role of GPER1, a tumor-promoting role has also been described under certain conditions of overexpression and specific histological subtypes. Hambach et al. found that GPER1 overexpression enhanced cell proliferation, migration, and stemness properties in cervical squamous cell carcinoma (CSCC) cell line SiHa, indicating a more aggressive phenotype, whereas an opposite effect was observed in the CAC cell line HeLa: reduced cell proliferation, migration, and increased cell apoptosis, all indicative of a less aggressive phenotype. Moreover, NGS analysis of the GPER1-overexpressing cells revealed that GPER1 oppositely regulated key cancer pathways, upregulating EMT, MYC, mTORC1, p53 and angiogenesis pathways in CSCC (SiHa cells), whereas, in CAC (HeLa cells), these pathways were downregulated together with KRAS, Hedgehog, TNFα (NFκB pathway) and Wnt/β-catenin [8].

Clinical analysis of GPER1 in CC shows a prognostic function depending on the stage and histological subtype underlying the complexity of this receptor in oncogenesis. In early stages of CC, high GPER1 expression has been consistently associated with better overall prognosis and relapse-free survival [3,8,98]. Also, this high GPER1 expression has been positively associated with tumor-suppression markers p16 and p53 [3,98].

Nevertheless, this general observation has been challenged by a critical divergence in the CAC subtype. Specifically, high GPER1 expression in CAC correlates with worse prognosis and invasive tumor growth in patients [3,8,82], thereby underscoring the need to distinguish CAC and CSCC as two different pathological entities in the context of hormonal signaling [8]. In CAC, E2-GPER1-mediated signaling regulates Claudin-1, a protein involved in tight-junction interactions [3,100]. This GPER1/Claudin-1 activation promotes malignant processes such as proliferation, migration, and invasion in CAC-derived cell lines, supporting the unfavorable prognosis of GPER1 overexpression in this histological subtype [82,100].

GPER1 also participates in other complex signaling mechanisms. GPER1 often transactivates EGFR (epidermal growth factor receptor) and its consequent downstream pathways [3,8,101]. High-risk HPV oncoproteins E6 and E7 were also reported to upregulate GPER1 mRNA and protein expression, while E7 modulates nuclear localization of GPER1 [3,102].

Furthermore, many estrogenic compounds have been shown to affect GPER1. MEHP (mono-ethylhexyl phthalate) is a xenoestrogen that promotes HeLa and SiHa cell proliferation through the GPER1/PI3K/Akt pathway without affecting invasion and MMP expression [3,103]. In SiHa cells, prolame induced cell proliferation, whereas butolame and pentolame showed no proliferative effects, even though molecular docking studies suggested that 17β-aminoestrogens interact with the hydrophobic cavity of GPER1. Finally, both E2 and 17β-aminoestrogens reduced c-fos phosphorylation in SiHa cells [101].

In summary, GPER1 is a prognostic factor and a key therapeutic target in CC [8,98]. However, its dual behavior underscores the critical need to distinguish histological subtype when selecting a therapeutic strategy since GPER1 modulation can promote or suppress oncogenesis in CSCC or CAC, respectively [8].

#### 5.1.6. Integrative View: Rewiring Estrogen Responsiveness in CC: Context, Compartment, and Plasticity

E2 signaling in CC is highly context-dependent during tumor progression. Rather than being driven by a single ER, CC involves a progressive reorganization of E2 responsiveness, including loss of epithelial ERα, persistence of ERβ, stromal ERα-dependent effects, and activation of alternative receptors such as ERα-36 and GPER1. These mechanisms allow estrogenic signaling to remain functionally relevant even when HPV-driven oncogenic pathways become dominant. Accordingly, E2 signaling in CC should be viewed as a modulatory network that contributes to invasion, metabolic adaptation, and therapeutic response in a stage- and histological subtype-dependent manner.

### 5.2. Ovarian Cancer

#### 5.2.1. Epidemiology and Clinical Challenges

With 324,603 cases and 206,956 deaths in 2022, ovarian cancer exhibits one of the worst incidence-to-mortality ratios among gynecological tumors [104]. Most cases are diagnosed at advanced stages, which contributes to their poor prognosis despite recent therapeutic advances [105,106]. Its etiology is multifactorial and involves genetic factors, particularly BRCA1/2, as well as hormonal and reproductive elements associated with the cumulative number of ovulatory cycles [107,108,109]. In addition, metabolic and lifestyle aspects play a role, whereas high parity, breastfeeding, and long-term use of combined oral contraceptives are consistently associated with a significant reduction in ovarian cancer risk [110,111,112].

#### 5.2.2. The ERβ-to-ERα Switch During Ovarian Carcinogenesis

##### The ERα/ERβ Switch: From Guardian to Villain

The ERβ (and its splicing variants) predominance in the normal ovary (Table 2) contrasts with OC, in which ERβ expression progressively decreases or is even lost during malignant transformation and tumor progression [41,113,114], whereas ERα becomes predominant, exhibiting a significant increase in the mRNA proportion of *ESR1/ESR2* in tumor tissue compared to normal ovary [38]. This switch in the ERα/ERβ balance is important for malignant transformation and proliferation since ERα is considered to be protumor, whereas ERβ acts as a tumor suppressor [34,40,70,115], a topic that will be addressed in more detail later in this review.

Meanwhile, ERα localization varies dynamically depending on the ovarian physiological state. In women of reproductive age, ERα is detected within the ovarian stroma and surface epithelium, as well as in the corpus luteum [34,116]. This latter localization suggests a regulatory role in postovulatory structures. On the other hand, in postmenopausal women, ERα is still present in the ovarian stroma, ovarian surface epithelium, and inclusion epithelial cysts [116]. This stromal persistence in postmenopausal women becomes important considering local estrogen (non-E2 estrogens) synthesis because, after cessation of ovarian function, peripheral tissues such as the liver, adrenal glands, brain, adipose tissue, and, crucially, ovarian stroma continue producing estrogen [117,118]. Moreover, estrogen production by peripheral tissues and the postmenopausal ovary is mediated by aromatase (CYP19), which catalyzes the conversion of androgens to estrogens [118,119,120]. Due to the stromal persistence of ERα [116], this tissue retains its ability to respond to estrogens produced in situ, which is fundamental for tissue homeostasis and the pathogenesis of hormone-sensitive cancers [119,120].

In ovarian cancer, ERα and ERβ co-expression is a well-established characteristic, although its prevalence varies depending on the histological subtype and the detection methodology used [119,121]. Previous studies have shown that ERα and ERβ were detected in a wide range of OC cases, from 60% to 100% of the cases [43]. However, recent studies indicate that ERα and ERβ are co-expressed in approximately 80% of patients with OC [9,121]. ERα is detected in 60% of OC cases [38] and in 80% of the most common and lethal subtype, high-grade serous ovarian carcinoma (HGSOC) [122]. Moreover, *esr1* mRNA transcripts were detected in about 60% of OC tissues [38]. Regarding ERβ, its expression is highly frequent in tumor tissue. Shafrir et al. analyzed 245 cases and found that 71% of the OC cases showed nuclear ERβ expression, whereas 43% of the cases exhibited cytoplasmic staining for ERβ [123]. Concerning ER expression in histological subtypes, in serous adenocarcinoma, ERα was detected in 97%, and 41% expressed ERβ, whereas, in endometrioid carcinoma, ERα was detected in 100% and ERβ was present in 75% of cases [38].

As mentioned above, it is important to note that, although both ERs are frequently co-expressed, disease dynamics are associated with a shift in the balance, in which the ESR1/ESR2 ratio (ERα/ERβ) significantly increases in OC tissue compared with the normal ovary [37]. For example, in OC cell lines co-expressing both receptors, ERα:ERβ ratios were high, such as 57:1 in SKOV3 and 14:1 in OV2008 [43]. This co-expression provides the basis for their functional interaction, which is crucial for tumor proliferation [43,124]. This ER dominance switch from ERβ to ERα is mainly due to epigenetic regulatory mechanisms leading to ERβ transcriptional silencing, and, in some contexts, to the compensatory/permissible activation of ERα [37]. During malignant progression, the gradual decrease or complete loss of ERβ represents a key molecular event [113,114]. The strongest evidence indicates that this process is mediated by DNA hypermethylation of *ESR2* promoter regions, resulting in gene repression and the consequent loss of ERβ expression in invasive OC cells [37,44]. These promoter regions are rich in GC sequences, which are particularly susceptible to DNA methyltransferase-mediated hypermethylation [125].

##### Epigenetic Reprogramming in OC: Silencing the Protector

Analysis of *ESR2*-specific promoter regions such as 0K and 0N in tumor tissues and malignant cell lines demonstrated that the 0N region is extensively methylated, correlating with the loss of ERβ1, ERβ2, and ERβ4 expression [37,44]. In addition to DNA methylation, ERβ reduction is associated with histone modifications that contribute to a chromatin-repressive state. In this context, treatment with HDAC (histone deacetylase) and DNMT (DNA methyltransferase) inhibitors showed a partial reversal of ERβ silencing in in vitro models, thus indicating that this is a dynamic and potentially reversible process resulting from the interaction between promoter hypermethylation and changes in histone acetylation [37,126].

In parallel, the relative dominance of truncated or malignant isoforms gains clinical relevance in advanced disease. ERβ2 and ERβ5 isoforms promote malignancy by enhancing cell migration, invasion, and proliferation [9,43,127]. The cytoplasmic localization of ERβ2 in advanced serous tumors predicts unfavorable clinical outcomes and has been linked to chemoresistance and inhibition of pro-apoptotic pathways [9,127]. Likewise, nuclear expression of ERβ5 is frequently observed in late-stage disease and correlates with adverse prognosis, particularly in serous and clear-cell carcinomas [9,127], but how can truncated ERβ isoforms, such as ERβ2 and ERβ5, gain functional relevance in OC despite the overall downregulation or epigenetic silencing of *ESR2*? As mentioned, hypermethylation of the *ESR2* 0N promoter mediates global downregulation of ERβ variants, including ERβ1, ERβ2, and ERβ4 [34,47], leading to loss of ERβ1 protective activity [41,113], but ERβ2 and ERβ5 remain functionally relevant because they mediate protumorigenic processes [9,43,127]. The functional dominance of ERβ2 and ERβ5 in OC arises not from high absolute expression levels but from their intrinsically malignant phenotype and the disruption of hormonal control caused by epigenetic silencing [41].

From a functional perspective, the loss of ERβ expression represents the loss of a key tumor-suppressive component in the ovary [113,115]. In contexts where both ERs are co-expressed, ERβ exerts an antagonistic effect on ERα-mediated transcriptional activity [124]; therefore, the removal of this molecular brake leads to ERα-mediated protumor processes such as proliferation, invasion, and EMT [38,41,121,128]. This functional shift is reflected in a significant increase in the *ESR1/ESR2* ratio in tumor tissue compared with normal ovarian tissue [37,41].

In parallel, high ERα expression observed in OC, particularly in HGSOC, is associated with *ESR1*-related epigenetic alterations [37]. As mentioned above, approximately 80% of these OCs express this receptor [38,122,129]. In this context, ERα hypomethylation has been identified as one of the main mechanisms that favors its overexpression in tumor tissues compared with normal tissue [37,130]. This ERα hypomethylation is associated not only with higher levels of ERα but also with better overall survival in patients with OC [130].

This observation appears counterintuitive given that ERα has been extensively linked to protumor signaling in ovarian cancer, raising the question of how *ESR1* hypomethylation and higher ERα levels correlate with better overall survival [130]. This apparent paradox can be explained by considering the functional role of ERα in the clinical context of OC. Mechanistically, ERα signaling suppresses homologous recombination repair by downregulating key DNA repair genes through cooperation with the corepressor CtBP, resulting in increased genomic instability. This defect in DNA repair enhances tumor sensitivity to platinum-based chemotherapy, which translates into a better treatment response and improved clinical outcome [131]. Therefore, high ERα expression reflects not only protumor signaling but also a tumor phenotype associated with increased chemosensitivity and favorable prognosis [37,131].

DNA demethylation leading to the transcriptional activation of *ESR1* is mediated by enzymes from the TET (ten–eleven translocation) family, responsible for removal of methyl groups from cytosines [59]. Similarly, transcriptional activity of ERα is regulated by epigenetic complexes such as the MegaTrans complex, which contains histone acetyltransferases and amplifies enhancer activity by forming super-enhancers enriched in activating marks like H3K27ac [37,132,133]. This epigenetic feedback loop contributes to the sustained activation of *ESR1*.

In contrast, *ESR1* promoter hypermethylation was also observed in certain contexts of OC, resulting in a reduction in ERα expression, indicating that its expression results from the balance between epigenetic activation and silencing mechanisms [37]. Regarding genetic mechanisms, point mutations of *ESR1* such as L536, Y537, and D538 were described. These mutations are associated with higher *ESR1* mRNA expression and with resistance to aromatase inhibitor therapy [134].

Together, in OC, the ERβ-to-ERα switch emerges as a direct consequence of coordinated epigenetic reprogramming, characterized by the silencing of the tumor suppressor ERβ and the sustained activation of the protumor ERα, thereby establishing a molecular context that promotes tumor progression [37,41].

Building on the ERβ-to-ERα switch observed during OC progression, the model of ER homo- and heterodimerization provides a clear mechanistic framework to explain changes in ER signaling. ER requires dimerization to function as a transcription factor, and ERα and ERβ can form homodimers or heterodimers upon ligand binding [9,124,135].

Direct evidence of ERα/ERβ heterodimer formation at the chromatin level in OC is still missing, a limitation acknowledged in the literature [131]. Most experimental evidence comes from non-ovarian models, such as breast cancer and osteosarcoma cell lines. In these models, ChIP-reChIP assays, inducible expression systems, and FRET-based biosensors have confirmed that ERα and ERβ can bind DNA simultaneously and interact in a ligand-dependent manner [42,135,136]. These studies demonstrate that ERα and ERβ have the molecular capacity to form functional heterodimers.

In OC, strong indirect evidence supports the functional relevance of ERα/ERβ interactions. When co-expressed, ERβ consistently antagonizes ERα-mediated transcription, acting as a molecular brake on ERα-driven protumor signaling [41,115,124,137]. This antagonism explains the tumor-suppressive role of ERβ observed in OC models, including reduced proliferation, invasion, and migration, and increased apoptosis following ERβ activation [43,138,139].

During malignant progression, epigenetic silencing and loss of ERβ lead to an increased ERα/ERβ ratio [38,41,114]. This shift removes ERβ-mediated repression, favoring ERα dominance and allowing unopposed ERα-driven transcriptional programs that promote proliferation, EMT, and cancer stem cell maintenance [128,137]. Therefore, even in the absence of direct genomic mapping in OC, the heterodimerization model (or, more broadly, ERβ-dependent antagonism of ERα) remains the simplest explanation for the functional consequences of the ERβ-to-ERα switch in OC [38,41].

#### 5.2.3. Functional Consequences of Estrogen Receptor Reprogramming in Ovarian Cancer

##### ERα Takes the Driver’s Seat

As previously mentioned, ERα functions as a protumor driver in OC, promoting proliferation, migration, invasion, EMT, and resistance to apoptosis [41,121,128]. ERα promotes tumor growth by inducing the expression of genes that are critical for proliferation and by activating non-genomic signaling pathways that inhibit apoptosis [121,128,140]. In vivo, E2 treatment increased tumor burden and promoted tumor growth in PEO4 xenograft models implanted in ovariectomized SCID/Beige mice [122]. Similarly, intraperitoneal injection of murine ascites cells (MASC1 and MASE2) into SCID mice, followed by implantation of E2 pellets, significantly reduced animal survival [141]. In vitro, E2 induced proliferation of Caov-3 and OVCAR-3 cells through ERα-mediated activation of the ERK and AKT pathways [128,140]. This effect was attenuated by shRNA-mediated ERα silencing, indicating that ERα is required for E2-driven proliferation, with ERK and PI3K–Akt signaling contributing to this response [140]. In addition, the ERα inhibitor MPP or the ERβ agonist DPN suppressed AKT phosphorylation in the SKOV3 cell line without affecting total AKT levels, suggesting that the protumor actions of ERα and the tumor-suppressive effects of ERβ converge on this pathway [43]. Consistently, treatment with fulvestrant, a more potent ERα inhibitor than tamoxifen, prevented E2-induced proliferation in PEO4 and PEO1 cells [122].

Beyond non-genomic signaling, ERα also promotes tumor progression through the transcriptional regulation of canonical target genes such as *GREB1*, *CCNG2*, and *MYC* [122,142]. Among these, *GREB1* has been identified as an early E2-responsive factor and a key regulator of estrogen-driven cell growth [141]. Microarray analysis of tumors derived from MASE2 ascites cells in E2-treated mice showed *GREB1* as one of the most upregulated genes. Functional studies demonstrated that shRNA-mediated knockdown of *GREB1* in MASE2 cells decreased cell proliferation in vitro and significantly extended the survival of xenograft-bearing mice in vivo [141].

ERα activation by E2 also promotes resistance to cisplatin-induced apoptosis in OC cells [140,143]. In Caov-3 and Ovcar-3 cells, E2 pretreatment followed by cisplatin exposure reduced PARP cleavage and increased the expression of the anti-apoptotic protein Bcl-2, thereby limiting apoptotic cell death. Mechanistically, cisplatin was shown to induce ERα phosphorylation at S118 through activation of the ERK pathway, leading to enhanced ERα transcriptional activity at ERE sites. Inhibition of MEK with PD98059 reduced cisplatin-induced ERα activation, whereas inhibition of the PI3K/Akt pathway with LY294002 had no effect, indicating that ERK signaling is the dominant pathway mediating ERα-dependent cisplatin resistance in these models. Taken together, the combined activation of ERα by E2 and cisplatin may contribute to platinum resistance in OC cells through increased expression of anti-apoptotic proteins such as Bcl-2 [140].

Experimental evidence also indicates that E2 promotes migration, invasion, and EMT in OC through ERα-mediated regulation of E-cadherin and EMT-associated transcription factors, including Snail and Slug [128]. In vitro studies using ERα-positive OC cell lines consistently show that ERα activation is sufficient to induce EMT-related phenotypes associated with increased tumor aggressiveness [137,144]. In OVCAR3 and PEO1 cells, activation of ERα with the selective agonist PPT increased the ALDH-positive cell population, a marker of cancer stem cells, commonly linked to EMT induction [137]. In the same experimental models, ERα activation enhanced cell migration and invasion in transwell assays, supporting a direct role of ERα in promoting EMT-associated functional changes [138]. At the molecular level, E2 was shown to regulate the transcription of Snail and Slug, leading to repression of E-cadherin expression and acquisition of metastatic potential, first demonstrated in OC models and later confirmed in the ERα-positive BG-1 cell line [137,144].

ERα signaling further contributes to invasion by regulating the remodeling of the extracellular matrix. In vitro exposure of OVCAR-3 cells to bisphenol A (BPA), an estrogenic compound acting through ERα, significantly increased cell migration and invasion, effects that were reversed by co-treatment with the ERα inhibitor MPP. This invasive behavior was associated with ERα-dependent upregulation of matrix metalloproteinases MMP-2 and MMP-9, enzymes required for extracellular matrix degradation during tumor invasion. In addition, ERα activation promoted metastatic dissemination by increasing the adhesion of OC cells to endothelial cells. BPA treatment enhanced the adhesion of OVCAR-3 cells to human umbilical vein endothelial cells (HUVEC), an effect linked to upregulation of intercellular adhesion molecule-1 (ICAM-1) and reversed by ERα inhibition [145]. Overall, these findings support a central role for ERα in EMT, invasion, and early metastatic steps in OC.

Altogether, ERα emerges as a major mediator of estrogen-driven oncogenic programs in OC, integrating genomic and non-genomic signaling pathways that support tumor progression and aggressiveness.

##### ERβ as Tumor Suppressor: The Lost Brake

On the other hand, and as previously mentioned, ERβ is widely recognized as a tumor suppressor in OC, its antitumoral role accomplished by several molecular mechanisms that negatively regulate cell growth, invasion, and malignant plasticity [41,115]. This tumor-suppressive effect of ERβ was demonstrated in vitro after ERβ overexpression reduced BG-1 cell proliferation, evidenced by a significant reduction in S phase and the modulation of pRB and cyclins D1 and A2 [138]. Similarly, DPN-mediated ERβ activation significantly suppressed SKOV3 and OV2008 cell growth, an effect that was synergistic with ERα inhibition [43]. This antiproliferative effect was partly achieved by inducing the cyclin-dependent kinase p21 (WAF1) and by reducing cyclin A2 mRNA levels after ERβ1 transfection of SKOV3 cells [139]. Moreover, treatment with DPN or MPP reduced Akt phosphorylation up to 80% in SKOV3 cells [43].

During metastasis, ERβ acts as a guardian of the epithelial phenotype by inhibiting migration, invasion, and EMT [128,137]. Experimental evidence has shown that ERβ re-expression in ovarian clear-cell adenocarcinoma inhibited migration and invasion [146]. A recent study using the selective ERβ agonist OSU-ERb-12 demonstrated that this compound suppresses EMT as it increases E-cadherin expression and reduces the expression of the Snail transcription factor, resulting in a reduction in PEO1 and OVCAR3 cell migration and invasion [137]. Moreover, natural agonists liquiritigenin and S-equol also showed a significant reduction in migration and invasion of ES2 and SKOV3 cell lines [147].

In addition to its antiproliferative function, ERβ has a direct impact on the OC stem cell (OCSC) population and its sensitivity to chemotherapy. ERβ1 is highly expressed in OCSCs enriched in ALDH^+^ (aldehyde dehydrogenase-positive) cells [148]. Treatment with the selective ERβ agonist OSU-ERb-12 reduced the OCSC population in cell lines and xenografts, inhibiting non-OCSC cell conversion to OCSC (dedifferentiation) by suppressing EMT [137]. The selective ERβ agonist LY500307 (erteberel) significantly reduced OCSC viability, self-renewal, and invasion capacities [148]. At the molecular level, treatment with LY500307 increased *CDKN1A/p21* and *FDXR* (p53-regulated gene) expression and induced apoptosis, evidenced by an increase in PARP cleavage and caspase 3 in OCSCs [148]. Therapeutic relevance was further confirmed in vivo, where LY500307-driven ERβ activation significantly attenuated the tumor-initiating capacity of OCSCs in murine orthotopic xenograft models [148]. Moreover, treatment with liquiritigenin and S-equol sensitized OC cells to cisplatin and paclitaxel therapy in vitro [147]. Additionally, in athymic mice, ERβ overexpression not only reduced ovarian xenograft growth but also reduced tumor cell content in metastatic sites and increased animal survival [138].

Altogether, the available evidence highlights the protective role of ERβ in OC and indicates that its reduced expression during tumor progression may favor aggressive and therapy-resistant phenotypes.

##### GPER1: The Non-Classical Player

Activation of the GPER1 represents a non-classical E2 signaling pathway that plays a complex role in the pathogenesis and progression of OC [3,9,128,129]. GPER1 is widely expressed in high-risk OC, as well as in normal ovarian tissue, where it mediates estrogenic signals through second-messenger systems and regulates physiological processes such as follicular maturation [129,149,150,151].

In the tumor context, multiple studies have demonstrated that GPER1 is broadly expressed in high-grade OC, with higher levels detected in advanced stages and recurrent disease, suggesting an association with disease progression [149,151,152]. However, its prognostic value remains controversial. Whereas several reports have linked elevated GPER1 expression to reduced overall survival and activation of protumorigenic pathways such as EGFR–Akt signaling [151,152,153], other studies have suggested tumor-suppressive effects in OC cell lines or have found no conclusive prognostic significance in clinical cohorts [129,154,155]. Notably, high GPER1 expression, particularly when combined with elevated Dkk2 levels, has been associated with improved overall survival in epithelial OC [156].

Mechanistically, GPER1-mediated signaling is rapid and non-genomic, initiated by binding of E2, other estrogens, or selective agonists such as G1 [128,129]. Receptor activation triggers cyclic AMP production, intracellular Ca^2+^ mobilization, and transactivation of the EGFR, leading to downstream activation of the PI3K/Akt and ERK/MAPK signaling cascades [9,128,157].

These pathways have been extensively characterized in OC cell lines, including SKOV3, OVCAR5, CAOV3, IGROV-1, and KGN, SKOV3 being among the most frequently used models due to the expression of both ERα and GPER1 [151,158,159]. In these systems, GPER1 activation by E2 or G1 significantly enhanced cell migration and invasion, effects that were almost completely reversed by the selective GPER1 antagonist G15, confirming receptor specificity [151,159].

At the molecular level, GPER1 activation has been linked to upregulation of the glycolytic enzyme 6-phosphofructo-2-kinase/fructose-2,6-bisphosphatase 3 (PFKFB3) and increased phosphorylation of focal adhesion kinase (FAK), both of which are key mediators of invasion and tumor progression in OC [151]. This GPER1–PFKFB3–FAK axis directly connects non-genomic E2 signaling with metabolic reprogramming and enhanced cell motility. In addition, GPER1 promotes invasive behavior by regulating matrix metalloproteinases, particularly MMP-9, facilitating extracellular matrix degradation [128,158].

Despite these protumoral effects, some studies have reported opposing outcomes regarding cell proliferation. In OC cell lines such as SKOV3 and IGROV-1, activation with G1 suppressed proliferation and induced G2/M cell-cycle arrest through inhibition of tubulin polymerization, indicating that GPER1 signaling outcomes may be highly context-dependent and influenced by cellular background and ligand concentration [128,158].

The therapeutic relevance of GPER1 has gained particular attention since tamoxifen, a selective ER modulator widely used in antihormonal therapy, functions as a GPER1 agonist. In SKOV3 cells, tamoxifen failed to inhibit E2-induced migration, promoting in turn cell motility, suggesting that GPER1 activation may counteract the beneficial effects of ERα antagonism and negatively influence responses to antiestrogen therapies. Consistently, pharmacological inhibition of GPER1 with G15 prevented E2-induced upregulation of PFKFB3, further supporting the central role of this pathway in OC aggressiveness [151].

**Figure 3:**
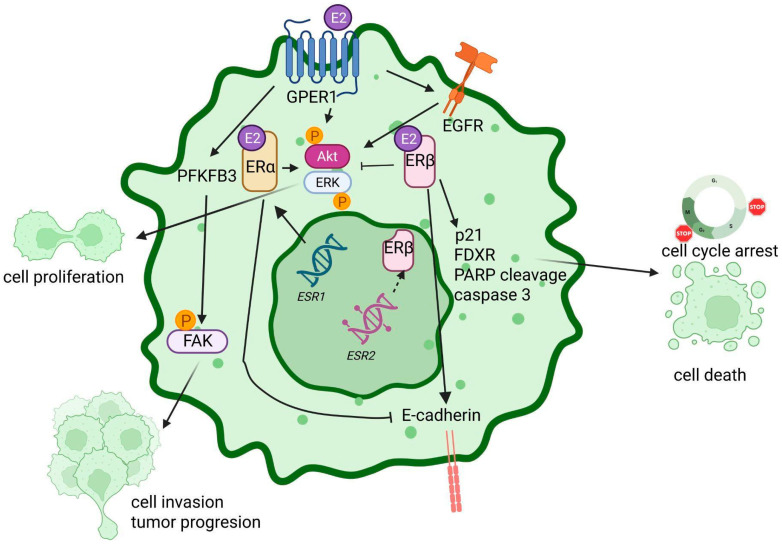
Role of estrogens and ERs in OC. OC cells express all estrogen receptors, with ERα predominating over ERβ, likely due to hypomethylation of the ESR1 promoter and hypermethylation of the ESR2 promoter. Overall ERβ expression is reduced, although the ERβ2 and ERβ5 isoforms are relatively increased; these localize to the nucleus and cytoplasm and are associated with poor prognosis. ERα promotes tumor cell proliferation and invasion through activation of the Akt and ERK pathways and downregulation of E-cadherin. In contrast, ERβ mainly exerts tumor-suppressive effects by inhibiting Akt and ERK signaling, inducing p21 and ferredoxin reductase (FDXR), promoting PARP cleavage and caspase activation, and enhancing E-cadherin expression, consistent with a more differentiated phenotype. GPER1 contributes to tumor progression via EGFR transactivation, leading to Akt and ERK activation, and by stimulating 6-phosphofructo-2-kinase/fructose-2,6-bisphosphatase 3 (PFKFB3), focal adhesion kinase (FAK) phosphorylation, and MMP9 expression, thereby supporting tumor growth and invasion. Figure created with BioRender.

Taken together, the high frequency of GPER1 expression in ovarian carcinoma and its involvement in multiple tumor-promoting processes have stimulated growing interest in the development of therapeutic strategies based on selective GPER1 antagonism. The role of ERs in OC is summarized in Figure 3.

#### 5.2.4. Integrative View: Dynamic Estrogen Signaling in OC

Overall, E2 signaling in OC is not driven by a single receptor but by a dynamic balance between ERα, ERβ, and GPER1. OC progression involves epigenetic silencing of the tumor-suppressive ERβ, increased dominance of ERα-mediated protumor signaling, and activation of non-classical E2 pathways through GPER1. The contribution of each receptor depends on tumor subtype, disease stage, and cellular context, which helps to explain the heterogeneous biological and clinical behavior of OC. This complexity highlights the importance of context-dependent approaches when considering E2 signaling as a therapeutic target in OC.

### 5.3. Endometrial Cancer

#### 5.3.1. Hormonal and Metabolic Determinants of Endometrial Cancer Risk

In 2022, nearly 98,000 deaths and about 420,000 new cases worldwide were registered for EC (uterine corpus cancer), ranking around 19th for mortality and 15th for incidence among all cancers. Incidence rates vary widely across regions, with higher age-standardized rates in high-income countries, while mortality remains lower but uneven worldwide, reflecting differences in risk factors, early diagnosis, and access to treatment [104]. Although many cases are diagnosed at an early stage, the global problem of EC is increasing, in parallel with population aging and the rising prevalence of metabolic disorders.

EC has a multifactorial etiology that is strongly related to hormonal and metabolic factors, with long-term exposure to unopposed estrogen playing a key role. Early menarche, late menopause, nulliparity, estrogen-only menopausal hormone therapy, and tamoxifen use, as well as obesity and type 2 diabetes, are reported as the main risk factors [160,161,162]. Concerning modifiable factors, excess body fat is the most important and consistently reported risk factor, acting through increased E2 production in adipose tissue, insulin resistance, and chronic low-grade inflammation [161,163]. In contrast, combined oral contraceptive use, parity, and breastfeeding are consistently associated with a reduced risk of EC [161].

EC is a highly heterogeneous disease that has been classically divided into Type I and Type II tumors, with distinct biological and clinical characteristics. Type I EC is predominantly endometrioid, E2-dependent, and generally associated with a favorable prognosis when diagnosed at early stages [164,165]. In contrast, Type II EC is considered E2-independent, encompasses aggressive histological subtypes such as serous and clear-cell carcinomas, and is typically associated with poor clinical outcomes [164]. At the molecular level, Type I EC frequently retains high ERα expression and is commonly characterized by alterations in the PI3K/PTEN pathway, whereas Type II EC often shows loss of hormone receptor expression and harbors TP53 mutations [166,167]. These fundamental differences underscore that ER signaling does not exert a uniform role across EC subtypes and must be interpreted within the specific histological and molecular context [166,167]. Although recent molecular classifications have further refined EC stratification, the Type I/Type II framework remains useful for contextualizing the divergent roles of ER signaling discussed in this review [166,167].

These observations point to a key role of E2 signaling in endometrial carcinogenesis. The endometrium is a hormone-responsive tissue in which E2 stimulates cell proliferation, whereas progesterone counteracts E2-driven growth; disruption of this balance favors tumor development [49,50]. Changes in ER expression and signaling are well documented in EC, underlying the importance of focusing on ER-mediated mechanisms in this disease [50,168].

#### 5.3.2. Reprogramming Estrogen Receptor α: From Endometrial Homeostasis to Tumor Autonomy

ERα evolution during endometrial carcinogenesis is a progressive process that depicts the passage from hormone regulation to an autonomous oncogenic role. This transition involves ERα structural modifications that sequentially alter intracellular signaling, transcriptional regulation, and tissue metabolism, allowing the endometrium to adapt—and eventually escape—physiologic control mechanisms characteristic of each pathological stage [9,49,50].

In healthy endometrium and the initial stages of endometrial hyperplasia (EH), the canonical ERα (66 kDa isoform) is the main mediator of E2 signaling [9,49], binding to ERE sequences in the nucleus and inducing the expression of genes involved in cell proliferation and survival, such as the progesterone receptor (PgR), cyclin D1, and Bcl-2 under tightly regulated physiological conditions [9,50,169].

In addition to classic genomic signaling, ERα participates in a bidirectional functional interaction with the insulin receptor (InsR-β). E2-induced InsR-β and IRS-1 phosphorylation amplifies the mitogenic stimuli of the PI3K/Akt and MAPK/ERK pathways [170]. This crosstalk becomes particularly relevant in altered metabolic contexts. In vitro studies conducted by Gu et al. using EC cell lines Ishikawa and RL95-2 demonstrated that hyperglycemia significantly increases ERα mRNA and protein levels, establishing a positive feedback loop between glycemic and estrogenic signaling. This ERα increase directly stimulates GLUT4 expression, promoting higher glucose intake and sustaining the energetic demands associated with deregulated glandular proliferation. The functional dependence of the ERα–GLUT4 axis on E2 signaling was confirmed by the use of specific ERα antagonists, which significantly reduced GLUT4 expression even under hyperglycemic conditions, validating the central role of this receptor in early metabolic reprogramming of endometrial tissue [171].

From an angiogenic point of view, glucose-induced ERα overexpression promoted VEGF secretion, thus facilitating the development of a new vascular network that is essential to support tissue expansion. In parallel, this active pathway activates TWIST and Snail, two key EMT regulators that reduce cell adhesion and favor a more invasive phenotype [171]. All these events are reinforced by insulin non-genomic actions that enhance ERα-mediated transcriptional activities by phosphorylation at S118 [170].

Studies conducted in vivo by Tian et al. using a nude mice xenograft model showed that combined treatment with E2 and insulin promotes significantly more aggressive tumor growth than treatment with any of these stimuli separately, confirming the metabolic–hormonal crosstalk in preneoplastic progression towards EC [170].

In preneoplastic stages, the importance of the canonical ERα role has been confirmed by IHC analysis performed by Hu et al. in a cohort of 125 human samples, including normal endometrium, atypical endometrial hyperplasia (AH), and EC [172]. In this study, ERα levels were the highest in atypical hyperplasia compared to those observed in normal endometrium, whereas, in EC, those levels began to decline. Together, these findings support the idea that canonical ERα acts as the main mitogenic engine during initial endometrial carcinogenesis, deregulating cell growth before the definitive malignant transformation [172].

During the transition from normal endometrium to hyperplasia, an ERα variant was detected, ERαΔ3, which is exclusively expressed in the preneoplastic stage. This isoform lacks exon 3, compromising the DBD domain, and arises from defective processing of *ESR1* mRNA. RT-PCR and sequencing analyses of human endometrial tissue demonstrated that ERαΔ3 is expressed in a stage-specific way as it was detected in most EH stages, while it was absent not only in normal endometrium but also in already established EC stages [50].

From a functional point of view, ERαΔ3 has a dominant negative effect on E2 signaling as its dimerization with canonical ERα is conserved, but the altered DBD domain efficiently abrogates binding to ERE sequences, thus interfering with ERα-mediated gene transcription. This interference hacks the receptor’s transcriptional fidelity and destabilizes hormonal control of the cell cycle that, under physiological conditions, is maintained by the E2/progesterone balance. In this context, ERαΔ3 expression was understood as a molecular marker of tissue stress and rupture of endometrial homeostasis, indicating the closure of an adaptive phase and generating a proper microenvironment for accumulating additional alterations that lead to definitive malignant transformation [50].

As EC progresses, particularly in high-grade tumors, a global decrease in ERα expression is observed. This phenomenon has been associated with more aggressive biological behavior and reduced dependence on circulating E2 stimulation [49,173]. In this context of tumor progression and dedifferentiation, ERα evolution culminates in the appearance of point mutations in the ligand-binding domain (LBD) of the *ESR1* gene, mainly affecting residues Y537 and D538. These mutations were identified by somatic DNA sequencing in a large cohort of 1034 EC patients, with an overall frequency of 1.8%, but were enriched in cases of advanced or recurrent disease. From a functional perspective, these mutations give the receptor constitutive activity, stabilizing it in an active conformation independent of E2 presence, thereby allowing continuous activation of transcriptional programs related to cell proliferation and survival [173]. From a clinical and metabolic point of view, these mutations were significantly associated with patients presenting a low body mass index, suggesting a metabolic bypass mechanism by which the tumor acquires proliferative autonomy without requiring the excess peripheral E2 typically associated with obesity, resulting in a more aggressive EC phenotype that is uncoupled from classical metabolic risk factors [49,173].

In summary, ERα demonstrates a stage-dependent evolution during endometrial carcinogenesis. From orchestrating controlled proliferation in normal and EH to generating transcriptional instability through isoforms such as ERαΔ3 and ultimately achieving ligand-independent activation via LBD mutations, ERα exemplifies how a hormone receptor can acquire autonomous oncogenic functions. This trajectory underscores its central role not only in driving early preneoplastic growth but also in facilitating the emergence of high-grade E2-independent tumors, emphasizing the need for precise stage-specific therapeutic strategies [49,50,173].

#### 5.3.3. ERβ Signaling in Endometrial Carcinogenesis: Loss, Rewiring, and Functional Paradox

The progression from EH to EC involves a progressive breakdown of estrogenic homeostasis, in which the functional loss of ERβ, particularly its canonical isoform ERβ1, represents a central event for the acquisition of proliferative autonomy. Unlike ERα, whose mitogenic role dominates the early phases of the disease, ERβ mainly acts as a negative modulator of E2 signaling, exerting tumor-suppressive functions through cell cycle control, apoptosis induction, and restriction of ERα-mediated transactivation [50,55,172]. The progressive loss of ERβ does not occur abruptly but rather follows a well-defined chronological sequence that parallels endometrial tissue dedifferentiation and precedes the establishment of malignant transformation [55,172].

The reduction in ERβ1 mRNA and protein levels during EH–EC progression is not associated with recurrent mutations in the *ESR2* gene but mainly relies on epigenetic silencing mechanisms [55,174]. Gene expression studies performed by Hojnik et al. in paired human samples demonstrated that total *ESR2* transcript levels are significantly decreased in tumor tissue compared to adjacent healthy endometrium in both premenopausal and postmenopausal women [55]. This transcriptional downregulation has been linked to hypermethylation of CpG islands located in the *ESR2* promoter region, a process mediated by increased activity of DNA methyltransferases, particularly DNMT1 and DNMT3B, which block the access of transcriptional machinery [50,55].

Available evidence supports DNMT1 and DNMT3B overexpression as a central epigenetic event during endometrial carcinogenesis; however, the upstream signals sustaining DNMT activation remain incompletely defined and are likely multifactorial. Human paired-sample transcriptomic and IHC analyses conducted by Hojnik et al. have consistently shown increased DNMT1 and DNMT3B expression in hyperplastic and malignant endometrium compared with adjacent normal tissue, in parallel with reduced *ESR2* mRNA and protein levels, in both premenopausal and postmenopausal women [55]. These observations raise the question of whether DNMT activation is primarily driven by prolonged estrogenic stimulation, functional ERα dominance, or metabolic stress [50,172]. Experimental models provide mechanistic support for all three contributors: E2 exposure alters DNMT expression and localization in endometrial stromal and epithelial cells [50]; hyperglycemic conditions upregulate ERα expression and sensitize cells to E2-driven proliferation [50,171]; and insulin signaling modulates the epigenetic landscape through TET–DNMT interactions within an inflammatory microenvironment [50]. Together, these data suggest a convergent model in which hormonal and metabolic cues cooperate to sustain DNMT activity during disease progression, although their relative contribution at different stages remains unresolved [50,55].

A second unresolved issue concerns the apparent selectivity of DNMT recruitment to the *ESR2* promoter [55]. Methylation-specific PCR and bisulfite sequencing analyses demonstrated early and robust hypermethylation of *ESR2* CpG islands during the transition from EH to EC, while other E2-responsive genes remain transcriptionally active at comparable stages [50,55]. This selectivity argues against random epigenetic drift and points toward a context-dependent targeting mechanism [55]. In vitro and tissue-based studies indicate that ERα-dominant signaling can promote DNMT recruitment via tethering to AP-1 and Sp-1 transcriptional complexes, particularly at loci involved in differentiation control, such as *ESR2* and *PGR-B* [50,175]. In parallel, loss of protective chromatin features—including FOXA1 binding and active histone marks—was reported at the *ESR2* locus under conditions of metabolic stress, which may further increase its vulnerability to epigenetic silencing [55,171].

Finally, the functional reversibility of *ESR2* promoter hypermethylation appears to be stage-dependent, raising the question of whether DNMT-mediated silencing acts as an initiating lesion or predominantly as a stabilizing mechanism [55]. Pharmacological DNMT inhibition using 5-aza-2′-deoxycytidine restores ERβ and progesterone receptor expression in EC cell lines and early-stage models, indicating that *ESR2* repression can be initially plastic and reversible [55,176]. In contrast, high-grade tumors exhibit near-complete loss of ERβ1 expression accompanied by the emergence of ERβ5, a splice variant lacking tumor-suppressive function that has been shown to cooperate with ERα to enhance oncogenic transcriptional programs [49,177]. ERβ5 importance will be further explained below. This transition is consistent with, but does not conclusively prove, a role for DNMT–spliceosome crosstalk, in which DNA methylation density and transcriptional elongation kinetics may bias alternative splicing toward oncogenic isoforms [49,55]. Collectively, these findings support the view that DNMT-mediated *ESR2* silencing can evolve from a reversible regulatory event into a mechanism that stabilizes a qualitative shift in E2 signaling during tumor progression [49,177].

From a functional perspective, ERβ1 expression represents a marker of endometrial epithelial differentiation. Its progressive loss reflects the dedifferentiation process characteristic of higher-grade tumors, being more pronounced in G2 and G3 carcinomas than in AH [55,172]. This dedifferentiation not only affects cell identity but also profoundly alters the architecture of E2 signaling by removing a critical component of ERα negative control [50].

At the molecular level, ERβ1 regulates the endometrial cell cycle through direct modulation of key genes involved in the G1–S transition [50,172]. Hu et al. conducted IHC analyses of human cohorts, including normal endometrium, AH, and EC, and showed that ERβ reduction inversely correlates with cyclin D1 levels and positively correlates with p21/WAF1/CIP1 expression [172]. ERβ1 promotes p21 expression, a cyclin-dependent kinase inhibitor that acts as a physiological brake on cell cycle progression, whose loss eliminates this critical checkpoint [50,172].

Functional models based on siRNA-mediated gene silencing in EC cell lines (Ishikawa, HEC-1A, and RL95/2) confirmed that experimental ERβ reduction accelerates the G1–S transition, increases proliferative rate, and reproduces the molecular pattern observed in human tumor tissues [172,178]. These effects are accompanied by sustained induction of cyclin D1 and activation of transcription factors associated with mitotic progression, thereby establishing a permissive environment for uncontrolled proliferation [172,174].

One of the most relevant findings associated with ERβ loss is the activation of the oncogene MYBL2 (B-MYB), a key regulator of S phase and mitosis. Quantification studies of ERβ variants in endometrial tissues demonstrated a positive correlation between ERβ1 decrease and MYBL2 upregulation, particularly in high-grade carcinomas [174,177]. ERβ knockdown assays in endometrial adenocarcinoma cell lines confirmed that ERβ reduction is sufficient to induce MYBL2 expression, accelerating cell proliferation and favoring tumor progression [174,177].

This functional link positions ERβ as an indirect negative regulator of MYBL2. Under physiological conditions, ERβ contributes to repression of this mitotic pathway; its loss releases a transcriptional program that promotes uncontrolled DNA replication and mitotic entry, reinforcing the aggressive phenotype of EC [172,174].

EC does not depend exclusively on absolute ERα or ERβ expression but on their functional balance. In normal endometrium, ERβ antagonizes ERα mitogenic activity through heterodimerization, competition for ERE binding, and differential recruitment of corepressors [50,172]. During progression toward carcinoma, ERβ1 reduction causes a marked increase in the ERα/ERβ ratio, leaving the tissue exposed to an “unopposed” estrogenic signaling context [55,177].

This imbalance potentiates activation of intracellular cascades classically associated with tumor growth, including MAPK/ERK and PI3K/Akt pathways, already described as central drivers in EC [174]. As mentioned above, ERα dominance favors induction of pro-proliferative genes, reduces sensitivity to apoptotic signals, and facilitates epithelial–mesenchymal transition, contributing to tumor invasiveness [50,55].

In advanced stages of endometrial carcinogenesis, progressive loss of the canonical tumor-suppressive isoform ERβ1 is accompanied by a qualitative remodeling of *ESR2* signaling rather than a simple quantitative depletion. Although total *ESR2* transcription remains globally repressed, alternative splicing of residual transcripts becomes selectively biased toward the ERβ5 variant, which is significantly enriched in high-grade tumors [55,177]. As mentioned, ERβ5 lacks the C-terminal transactivation domain required for classical ERβ-mediated transcription but retains the ability to heterodimerize with ERα, thereby modifying ER signaling output [49,177].

This selective emergence of ERβ5 in the background of overall ERβ depletion represents a biological paradox in EC: while the tumor progressively eliminates the growth-restraining functions of ERβ1, it preserves and enriches an isoform that functionally cooperates with ERα [174,177]. In this ERα-dominant context, ERβ1 loss and selective ERβ5 enrichment may together release cell-cycle restraint and potentiate ERα-driven proliferative and survival pathways associated with tumor aggressiveness [174,175,177]. Consistently, ERβ5 expression positively correlates with MYBL2 and HER2 levels in human tumor tissues, supporting its active contribution to mitotic progression and oncogenic transcriptional programs [174,177].

From a mechanistic perspective, this qualitative shift integrates multiple regulatory layers. Loss of ERβ1 removes transcriptional control over cell-cycle regulators such as cyclin D1 and p21, facilitating G1–S transition [50,172], while ERα/ERβ5 heterodimerization enhances cellular sensitivity to E2 and reinforces ERα-dependent signaling cascades. Together with DNMT-mediated stabilization of *ESR2* repression, these processes consolidate a functional reprogramming of E2 signaling toward a proliferation-permissive and hormonally hypersensitive state rather than restoring physiological ERβ-mediated homeostasis [49,55].

#### 5.3.4. GPER1 Signaling in Endometrial Cancer: Epithelial–Stromal Divergence

Evidence from human endometrial samples indicates that GPER1 expression progressively increases during the transition from normal proliferative endometrium to EH, rising from simple hyperplasia and reaching the highest levels in complex hyperplasia [50]. This increase parallels ERα expression during preneoplastic stages, suggesting coordinated regulation of both receptors in E2-responsive tissue. Mechanistically, GPER1 signaling has been proposed to contribute to hyperplastic progression through induction of aromatase expression, resulting in enhanced local E2 biosynthesis and sustained E2-driven proliferative signaling within the endometrium [50]. At this stage, GPER1 acts mainly as an amplifier of classical E2 signaling rather than an independent oncogenic driver, reinforcing MAPK/ERK activation and supporting glandular expansion [49,50].

In EC, IHC and clinical studies revealed that GPER1 is frequently overexpressed in high-risk tumors and negatively correlates with progesterone receptor expression [50]. High GPER1 levels are associated with advanced FIGO stage, high histological grade, deep myometrial invasion, and reduced overall survival [3]. The functional relevance of these associations has been demonstrated by Li et al. in experimental models. In SPEC-2 cells, autocrine motility factor (AMF) directly binds GPER1, activating PI3K/Akt signaling, promoting proliferation, and modulating apoptosis [50,179]. In nude mouse xenografts, silencing of GPER1 abolished AMF-induced tumor growth and significantly prolonged survival, supporting a tumor-promoting role for GPER1 signaling under these conditions [179].

Metabolic regulation further shapes GPER1 activity in EC. Analyses of biopsies from insulin-resistant EC patients showed high GPER1 expression concomitant with elevated levels of the epigenetic regulator TET1 [50]. In vitro experiments demonstrated that insulin induces TET1 expression via PI3K/Akt signaling, increasing DNA hydroxymethylation at the GPER1 promoter and enhancing its transcription [50,174]. This epigenetic regulation positions GPER1 as a sensor integrating systemic metabolic signals with E2 responsiveness in EC cells. Additional growth factor crosstalk has been described. In Ishikawa and KLE cells, IGF-1 transactivates the GPER1 promoter through the IGF-IR/PKCδ/ERK/c-fos/AP1 pathway, further reinforcing the convergence of growth factor and E2 signaling at the GPER1 level [50].

Strikingly, subcellular localization studies revealed that GPER1 is not restricted to plasma membrane but is also detected in endoplasmic reticulum, Golgi apparatus, and nucleus in EC tissues. These observations support the concept that GPER1 does not exist as distinct receptor entities but rather as functional states dictated by post-translational modifications and intracellular trafficking. Experimental evidence indicates that lack of N-terminal N-glycosylation redirects GPER1 from its canonical membranous localization to the nucleus [3,55]. Whereas glycosylated membranous GPER1 mediates rapid non-genomic E2 signaling—such as calcium mobilization, cAMP production, and EGFR transactivation—the non-glycosylated receptor acquires nuclear functions associated with transcriptional regulation [3,50]. A critical shift in GPER1 function emerges within the tumor microenvironment. In cancer-associated fibroblasts (CAFs), nuclear localization of non-glycosylated GPER1 was shown to promote expression of connective tissue growth factor (CTGF) [3]. CTGF secreted by CAFs acts in a paracrine manner on neighboring epithelial tumor cells, enhancing migration and invasion [3,50].

In addition to transcriptional effects, cytoplasmic GPER1 signaling in CAFs activates the cAMP/PKA/CREB pathway, inducing metabolic reprogramming toward increased glycolysis [3]. This stromal metabolic activation provides energetic substrates to tumor cells and contributes to resistance to therapies, such as tamoxifen and doxorubicin [3,180]. Together, these findings identify stromal GPER1 as an active architect of an invasive and therapy-resistant microenvironment.

In contrast to its consistently pro-invasive role in CAFs, GPER1 exhibits paradoxical behavior in tumor epithelial cells. In EC cell lines such as RL95-2 and HEC-1A, treatment with the selective GPER1 agonist G-1 reduced cell growth in a dose-dependent manner and induced apoptosis and cell cycle arrest [55,174]. These findings suggest that, in specific epithelial contexts, GPER1 can function as a non-genomic brake on proliferation. On the other hand, clinically, loss of epithelial GPER1 expression has been associated with advanced FIGO stage, higher histological grade, and progression toward metastatic disease [3,55]. In this setting, GPER1 appears to act as a marker of epithelial differentiation, whose absence facilitates dedifferentiation and epithelial–mesenchymal transition. Thus, the same receptor can exert protective or deleterious effects depending on cell lineage and subcellular localization. While epithelial GPER1 may constrain tumor progression under defined molecular conditions, stromal GPER1 consistently promotes invasion, matrix remodeling, and tumor–stroma crosstalk [3,179].

Pharmacological studies further support the contextual complexity of GPER1 signaling. In endometrial cells, tamoxifen acts as a GPER1 agonist despite antagonizing nuclear ERα, activating EGFR transactivation and ERK/cyclin D1 signaling and stimulating proliferation [3,50]. Similar GPER1-mediated mitogenic effects have been reported for fulvestrant and raloxifene [50]. In vivo relevance is supported by xenograft studies showing that activation of GPER1 promoted tumor formation from RL95-2 cells, whereas treatment with the selective antagonist G-15 delayed EC growth [50,174].

Post-transcriptional regulation also contributes to GPER1 variability during EC progression. MicroRNA-195 has been identified as a negative regulator of GPER1 expression in EC cell lines such as AN3-CA and HEC-1A, suggesting that disruption of microRNA networks may underlie either GPER1 overexpression or loss depending on tumor context [50].

Taken together, the available evidence supports a dynamic model of GPER1 function along the endometrial disease continuum. During hyperplasia, GPER1 primarily amplifies ERα-dependent E2 signaling through aromatase induction and MAPK activation [50]. In established EC, GPER1 integrates metabolic and growth factor cues, modulating tumor identity and E2 sensitivity via insulin–TET1 and IGF-1 signaling axes [50,170]. At invasive stages, GPER1 facilitates tumor progression through AMF-driven PI3K/Akt activation in tumor cells and nuclear non-glycosylated GPER1 signaling in CAFs, which promotes CTGF expression and stromal remodeling [3,179].

Overall, GPER1 cannot be classified as a conventional oncogene or tumor suppressor. Instead, it functions as a contextual signal transducer whose biological output depends on metabolic status, receptor network balance, cell lineage, and subcellular localization [3,50]. This plasticity has important implications for therapeutic targeting, indicating that strategies aimed at GPER1 must consider both tumor cells and the stromal compartment.

Within the proposed functional continuum—from E2-driven EH to metabolically integrated carcinoma and stroma-assisted invasion—GPER1 signaling appears not as a fixed oncogenic driver but as an ER program that depends strongly on the cell type and microenvironment [3,50]. This raises an important question for ER biology across cancers: does GPER1-mediated stromal activation represent a final irreversible state of the tumor microenvironment or a dynamic and potentially reversible program?

In EC, the pro-invasive activity of GPER1 in the stroma depends on regulatory layers—post-translational modifications, intracellular trafficking, and epigenetic-metabolic regulation—that are naturally flexible and not caused by permanent genetic changes [3,181]. Specifically, nuclear localization of the receptor, caused by the lack of N-terminal N-glycosylation, allows GPER1 to act in the nucleus and promote the expression of CTGF, a key factor in stromal-driven invasion [3,50]. Because this change comes from protein processing and not mutations, stromal GPER1 signaling could, in theory, be reprogrammed. Also, GPER1 expression and activity in the stroma are influenced by the body’s metabolic state. In patients with insulin resistance, TET1 is induced via PI3K/Akt, which increases hydroxymethylation of the GPER1 promoter and maintains its high expression, making GPER1 a sensor that connects metabolic signals with local E2 response [50,170]. Since this regulation is epigenetic and reversible, it suggests that correcting metabolism or blocking related signaling pathways could adjust stromal GPER1 activity [49]. Looking at it this way, stromal GPER1 activation fits with new ideas in hormone-dependent tumors, where stroma is not passive but actively regulates E2 signaling, therapy resistance, and invasion [3,181]. GPER1 works as a central node where E2 and metabolic signals meet in the tumor microenvironment.

**Figure 4:**
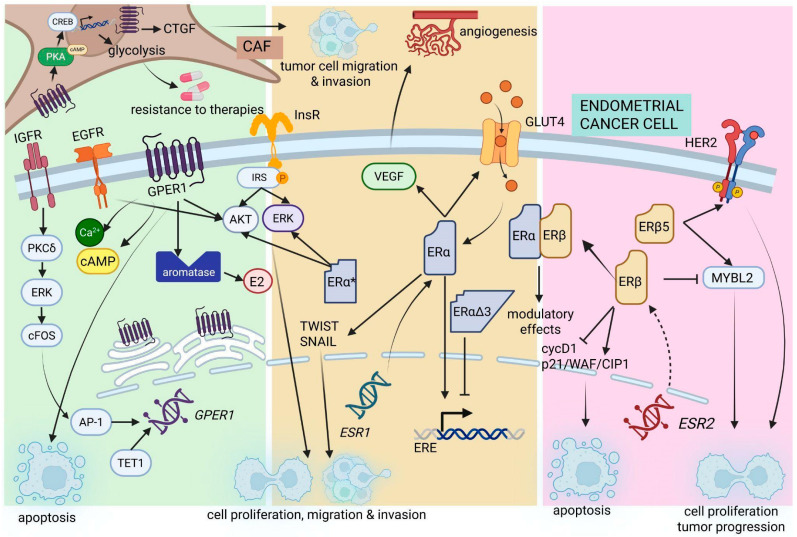
Summary of estrogen receptor (ER) actions in endometrial cancer (EC). (**Left**): Cancer-associated fibroblasts (CAFs) express GPER at plasma membrane and in the nucleus, where it promotes glycolytic gene expression, contributing to therapy resistance, and upregulates connective tissue growth factor (CTGF), enhancing tumor invasion and migration. In EC cells, GPER1 localizes to the plasma membrane, endoplasmic reticulum, and Golgi apparatus. IGFR/AP-1 signaling induces GPER1 expression, which is maintained by TET1-dependent hydroxymethylation. GPER1 stimulates aromatase expression, ensuring local E2 production, and transactivates EGFR, leading to Akt and ERK activation. GPER-mediated calcium mobilization and cAMP production further enhance E2 signaling, although, in specific cellular contexts, GPER activation may induce apoptosis. (**Center**): ERα activation induces VEGF and GLUT4 expression, promoting angiogenesis and glucose uptake, and upregulates TWIST and Snail, facilitating epithelial–mesenchymal transition (EMT) and cell migration. Constitutively active ERα* signals through Akt and ERK pathways. ESR1 expression is initially high in EC, whereas expression of the truncated ERαΔ3 isoform in preneoplastic stages impairs ERα transcriptional activity and favors malignant transformation. (**Right**): ERβ exerts tumor-suppressive effects by modulating ERα activity, downregulating cyclin D1 and MYBL2, and inducing cell cycle inhibitors such as p21. During tumor progression, ERβ is silenced by promoter hypermethylation; however, retention of the ERβ5 isoform sustains HER2 and MYBL2 signaling, supporting cell proliferation. Figure created with BioRender.com.

The summarized actions of ERs in EC are depicted in Figure 4.

Overall, these results suggest that GPER1 should not be seen only as a cell-intrinsic factor or a classic oncogene/tumor suppressor. Instead, it is a contextual signal transducer whose role depends on subcellular location, cell type, and metabolic state [3,50]. This view connects different E2-dependent cancers and highlights the importance of considering both tumor cells and stroma when designing therapies targeting ERs.

#### 5.3.5. Integrative View: Context-Dependent Estrogen Receptor Signaling in Endometrial Carcinogenesis

EC can be seen as a step-by-step change in ER signaling, where each receptor—ERα, ERβ, and GPER1—has its own role but also works together across the disease stages from normal tissue to cancer. ERα is the main driver of cell growth in early hyperplasia, working through both genomic and non-genomic pathways, and increasing proliferation and metabolism [9,49,50]. ERβ, especially the canonical ERβ1, acts as a brake, limiting ERα-induced proliferation, controlling the cell cycle, and keeping epithelial cells differentiated [50,55,172]. Loss of ERβ1 through DNMT-mediated epigenetic silencing and the appearance of ERβ5 change the E2 response, leaving ERα unchecked, activating oncogenes like MYBL2 and HER2, and reducing normal cell regulation [49,174,177]. GPER1 also plays a flexible role: in hyperplasia, it supports ERα-dependent growth, but, in cancer, especially in stromal fibroblasts, it assists invasion, changes metabolism, and contributes to therapy resistance [3,50,179].

Altogether, this shows that endometrial cancer is not caused by one receptor alone but by a network of ERs influenced by hormones, epigenetics, and metabolism [49,50,55]. How much of each receptor is present, which isoforms exist, where they are inside the cell, and the tissue environment all affect the switch from normal E2 signaling to uncontrolled cancer growth [3,174,179]. We need to look at both the amount and type of ER signaling to understand tumor start, progression, and possible treatments [50,177]. ERs do not just make tumors grow; they carry a context-dependent E2 signal that changes with disease stage, pointing to mechanisms and targets for therapy [49,50,55].

## 6. The Molecular Crossroads of Estrogen Signaling in Gynecological Cancers: Regulation by Non-Coding RNAs

Non-coding RNAs have emerged as critical post-transcriptional regulators of hormone-dependent signaling networks in gynecological tissues [182]. In the context of estrogen signaling, ncRNAs contribute to receptor plasticity by fine-tuning receptor expression, localization, and downstream responses in both physiological and pathological conditions. Non-coding RNAs (ncRNAs), including microRNAs (miRNAs) and long non-coding RNAs (lncRNAs), are key regulators of estrogen signaling in gynecological cancers [183]. Several miRNAs, such as miR-206 and members of the miR-200 family, directly modulate ERα and GPER1 expression, thereby influencing EMT, cell migration, and metastatic potential [151,183,184,185,186]. Depending on tissue context and hormonal status, these miRNAs may act as tumor suppressors or oncomiRs and can also serve as exosomal biomarkers of E2 responsiveness [184,185,186].

In EC and OC, estrogen-responsive lncRNAs, including ElncRNA1, SRA, and HOTAIR, reinforce estrogen-driven transcriptional programs that promote proliferation and invasion [187,188,189,190]. Several lncRNAs function as competing endogenous RNAs (ceRNAs), sequestering tumor-suppressive miRNAs and indirectly sustaining oncogenic signaling. A representative example is the LINC00899/miR-944/ESR1 axis in CC, where loss of ERα—frequently associated with HPV E7 activity—contributes to tumor aggressiveness [191].

Regulation of GPER1 by ncRNAs (e.g., miR-424 and miR-195) is emerging as a relevant mechanism in endocrine resistance, whereas ncRNA-mediated control of ERβ remains incompletely characterized [99,192]. These interactions are summarized in Table 3.

Collectively, ncRNAs form a complex regulatory network that controls ER signaling dysregulation in gynecological cancers. MiRNAs directly target ER isoforms, while lncRNAs influence ER transcription, receptor coactivation, or miRNA availability through ceRNA mechanisms. Although ERβ is generally considered to be tumor-suppressive, evidence of its regulation by ncRNAs remains limited in gynecological tumors, making it an important area for future research. From a translational standpoint, targeting ncRNA–ER regulatory circuits offers promising opportunities to overcome endocrine resistance and improve hormone-based treatments. Combined strategies using ER modulators with ncRNA-based therapeutics may ultimately improve precision medicinal approaches in hormone-responsive gynecological cancers.

## 7. Conclusions and Future Perspectives

Accumulating evidence demonstrates that E2 signaling in gynecological cancers cannot be adequately explained by a simple model based only on the presence or absence of ERs. Instead, E2 responsiveness reflects a dynamic and context-dependent network influenced by receptor subtype balance, isoform diversity, cellular localization, epigenetic regulation, and interactions with the tumor microenvironment [3,9].

In CC, E2 signaling remains functionally relevant despite the progressive loss of epithelial ERα expression. This persistence depends on stromal ERα-mediated paracrine signaling, the oncogenic activity of alternative ERα isoforms such as ERα-36, the sustained expression of ERβ in neoplastic cells, and the activation of non-classical ERs, including GPER1 [3,74,83,87]. Together, these mechanisms allow E2 to influence tumor progression, invasion, metabolic adaptation, and response to therapy, even in the presence of strong HPV-driven oncogenic signaling. The context-dependent and sometimes contradictory roles of GPER1 further highlight the importance of considering tumor subtype and disease stage when evaluating ER-based interventions [8,98].

In OC, malignant transformation is associated with strong reprogramming of ER signaling, characterized by epigenetic silencing of the tumor-suppressive ERβ and the relative predominance of ERα [37,41,44]. This ERβ-to-ERα shift removes an important regulatory constraint on E2-driven transcription, promoting proliferation, EMT, cancer stem cell maintenance, and metastatic behavior [115,137]. At the same time, high ERα expression is associated with specific biological vulnerabilities, including impaired homologous recombination repair and increased sensitivity to platinum-based chemotherapy, underscoring the context-dependent nature of E2 signaling in ovarian tumors [130,131].

In EC, E2 signaling evolves from a tightly regulated hormone-dependent process toward a more autonomous oncogenic program. The early stages are driven mainly by canonical ERα-mediated genomic signaling resulting from prolonged exposure to unopposed E2. As the disease progresses, tumor cells display truncated ERα isoforms, activation of non-genomic signaling pathways, metabolic reprogramming, and extensive crosstalk with insulin and inflammatory signaling pathways [9,49,50]. Concurrent changes in ERβ and GPER1 expression further contribute to uncontrolled proliferation, survival, and invasion, supporting the idea that endometrial carcinogenesis involves qualitative changes in ER signaling rather than estrogen exposure alone [168,169].

Across these malignancies, a common principle emerges: ER function is not determined only by receptor identity but also by isoform composition, subcellular localization, epigenetic regulation, ncRNA-mediated control, and interaction with other oncogenic pathways. This complexity helps to explain the apparent contradictions reported in the literature regarding ER expression, prognostic value, and therapeutic relevance.

From a translational perspective, these findings argue against uniform anti-estrogen strategies and support the development of more precise approaches tailored to tumor type, receptor profile, and disease stage. Future therapies may benefit from selectively targeting pathogenic receptor isoforms, modulating epigenetic mechanisms to restore protective E2 signaling, or exploiting context-dependent vulnerabilities created by ER reprogramming.

Despite the progress described in this review, several important questions remain open. We still do not clearly know when E2 signaling becomes oncogenic during tumor progression, whether this change happens gradually or abruptly, or if it can be reversed. It is also unclear which cell populations and subcellular compartments are mainly responsible for E2-driven oncogenic signaling in vivo, and how the expression of different ER isoforms is regulated over time. In this context, a major unresolved gap concerns the contribution of non-coding RNAs, including miRNAs and lncRNAs, to the dynamic regulation of ER signaling. While multiple ncRNAs have been implicated in the modulation of ER expression, localization, and downstream signaling, their integration into coherent tumor-specific regulatory networks remains poorly defined. In addition, the role of GPER1 remains controversial and appears to depend strongly on tumor type, disease stage, and cellular context. Addressing these unresolved issues will require integrative approaches combining spatial and single-cell analyses with functional studies and clinical correlation. Improving our understanding of when, where, and how E2 signaling is rewired is essential to move beyond descriptive receptor expression and toward more effective and context-aware endocrine strategies in gynecological cancers.

## Figures and Tables

**Table 1:** ERβ isoforms and their main characteristics.

Isoform	C-Terminal Modification	Estrogen Binding	Transactivation	Dimerization	Functional Role	References
ERβ1	None (full length)	Yes	Yes	Yes (α/β)	Active receptor	[23]
ERβ2	AF-2 disrupted	No	No (dominant negative)	Yes	Inhibitory	[24,25]
ERβ3	Truncated LBD	No	Unknown	Yes	Likely modulatory	[5]
ERβ4	Partial LBD	No	Weak	Yes	Modulatory	[5]
ERβ5	Truncated LBD	No	Coactivates ERβ1	Yes	Enhancing	[25,26]

**Table 2:** Estrogen receptors in normal gynecological tissues.

Tissue	Receptor	Menstrual Cycle Phase/Premenopausal State	Postmenopausal State	References
Cervix	ERα	Cyclical fluctuation according to estradiol levels	Marked decrease	[34]
	ERβ	Positive during follicular phase; negative during luteal phase	Constitutive expression (basal and parabasal layers)	[34,35]
	GPER1	Localized in epithelial layers; involved in epithelial proliferation and mucosal homeostasis	Present; reduced functional activity due to ligand deprivation	[28,56]
Ovary	ERα	Localized in theca cells and surface epithelium	Limited basal expression in stroma/theca	[9,34]
	ERβ	Abundant in granulosa cells (follicular development)	Predominant (nuclear); homeostatic and metabolic regulator	[9,36,38,45]
	GPER1	Constant distribution; involved in follicular maturation	Rapid signaling (calcium, ERK, PI3K/Akt)	[9,11]
Endometrium	ERα	Peak expression during late proliferative/early secretory phase	Significantly decreased expression	[50,51,52]
	ERβ	Peak during late secretory phase (stromal predominance)	Increased mRNA levels in myometrium	[36,50,51,52]
	GPER1	Maximum expression in glandular epithelium (mid proliferative phase)	Present in epithelium and stroma	[50,54]

**Table 3:** Non-coding RNAs targeting estrogen receptors in gynecological cancers.

ncRNA Type	Name	Functional Role	Tissue	Main Function	References
miRNA	miR-206	Tumor suppressor	Ova-ry/endometrium	Directly targets ERα; inhibits proliferation and migration	[151,183,193]
miRNA	miR-200 family	Context-dependent	Ova-ry/endometrium	Regulates EMT; expression influenced by ERα; exosomal biomarker	[184,185,186]
miRNA	miR-107-5p/miR-222-3p	OncomiRs	endometrium	Promote proliferation and invasion via ERα repression	[194,195]
miRNA	miR-21	OncomiR	Cervix	Promotes tumor growth via TIMP3/RECK axis; enhanced by HPV E7	[196,197]
miRNA	miR-424/miR-195	Tumor suppressors	Endometrium	Directly target GPER1; reduce proliferation and EMT	[99,192]
lncRNA	ElncRNA1	Oncogenic	Ovary	Estrogen-induced; promotes proliferation via feedback loop	[187,188]
lncRNA	SRA (SRA1)	Oncogenic	Ovary	Facilitates EMT and invasion;	[189]
lncRNA	HOTAIR	Oncogenic	Endometrium	Scaffold for E2-induced metastasis (miR-646/NPM1 axis)	[190]
lncRNA	NR2F1-AS1	Oncogenic	Endometrium	ceRNA mechanism; sponges miR-363 to release SOX4	[198]
lncRNA	LINC00899	Tumor suppressor	Cervix	ceRNA for miR-944; restores ERα expression	[191]

## Data Availability

Data sharing is not applicable to this article, as no new datasets were generated or analyzed in this study. This review is based on a curated selection of literature retrieved from the PubMed database; however, due to space limitations, not all relevant publications could be included. In addition, the literature search was limited to works published up to December 2025; therefore, studies appearing after this date are not covered in this review.

## Abbreviations

The following abbreviations are used in this manuscript:

17β-HSD1	17β-hydroxysteroid dehydrogenase 1
AF-1 and AF-2	activation functions -1 and -2
AMF	autocrine motility factor
BORIS	brother of regulator of imprinted sites
CAF	cancer-associated fibroblast
CC	cervical cancer
ceRNA	competing endogenous RNA
DBD	DNA-binding domain
DNMT	DNA methyltransferase
E1	estrone
E2	17β-estradiol
E3	estriol
E4	estetrol
EC	endometrial cancer
EMT	epithelial-to-mesenchymal transition
ER α/β	estrogen receptor alpha/beta
ERE	estrogen response element
FSH	follicle-stimulating hormone
GnRH	gonadotropin-releasing hormone
GPER1	G protein-coupled estrogen receptor 1
HDAC	histone deacetylase
HMGA2	high-mobility-group A2 protein
HPO	hypothalamic–pituitary–ovarian
HPV	human papillomavirus
IHC	immunohistochemistry
InsR	insulin receptor
LBD	ligand binding domain
LH	luteinizing hormone
MMP	metalloprotease
OC	ovarian cancer
PFKFB3	6-phosphofructo-2-kinase/fructose-2,6-biphosphatase 3
PgR	progesterone receptor
SIX1	sine oculis-related homeobox 1 homologue
